# Distance–resilient conductivity in p-doped polythiophenes

**DOI:** 10.1039/d5mh00620a

**Published:** 2025-08-25

**Authors:** Eva Röck, Demetra Tsokkou, Basil Hunger, Maximilian M. Horn, Sepideh Zokaei, Renee Kroon, Jesika Asatryan, Jaime Martín, Christian Müller, Martijn Kemerink, Natalie Banerji

**Affiliations:** a Department for Chemistry, Biochemistry and Pharmaceutical Sciences, University of Bern Freiestrasse 3 3012 Switzerland natalie.banerji@unibe.ch; b Department of Chemistry and Chemical Engineering, Chalmers University of Technology 412 96 Göteborg Sweden christian.muller@chalmers.se; c Laboratory of Organic Electronics, Linköping University SE-581 83 Linköping Sweden; d Universidade da Coruña, Campus Indistrial de Ferrol CITENI, Campus de Esteiro S/N 15403 Ferrol Spain; e Institute for Molecular Systems Engineering and Advanced Materials, Heidelberg University Im Neuenheimer Feld 225 69120 Heidelberg Germany martijn.kemerink@uni-heidelberg.de

## Abstract

Scalable organic electronic devices necessitate effective charge transport over long distances. We assess here the conductivity and its distance–resilience in doped polythiophene films with alkyl and oligoether side chains. We find that the polymers with oligoether side chains retain 80–90% of the conductivity over five orders of magnitude in distance (from tens of nanometers to millimeters), when doped with 2,3,5,6-tetrafluoro-tetracyanoquinodimethane (F_4_TCNQ). For P(g_4_2T-T) co-processed with F_4_TCNQ, this leads to an over 100 times enhanced long-range conductivity (43 S cm^−1^) compared to doped poly(3-hexylthiophene) (P3HT, 0.2 S cm^−1^). Optimization of the oligoether side chain length and doping protocol pushes the conductivity to 330 S cm^−1^. Kinetic Monte Carlo simulations of nanoscale terahertz conductivity data reveal that the local mobility of the doped P(g_4_2T-T):F_4_TCNQ film benefits from a higher dielectric constant (reduced Coulomb binding to the ionized dopant) and from lower energetic disorder. Those benefits persist on the macroscopic scale, while spatial charge confinement and a lack of connectivity hinder the long-range transport of moderately doped P3HT:F_4_TCNQ. However, strongly doping P3HT using magic blue leads to enhanced conductivity with distance-resilience >80%. The distance–resilience is generalized for different polymer:dopant systems once a highly conductive regime (>30 S cm^−1^) is reached. This highlights an effective strategy to overcome limitations in terms of electrostatic binding and multi-scale polymer ordering, enhancing both the short-range and the long-range conductivity of doped conjugated polymers.

## Abstract 2

Polythiophenes with oligoether side chains retain 80–90% of their conductivity over five orders of magnitude in distance (from tens of nanometers to millimeters), when doped with F_4_TCNQ.

## Graphical abstract

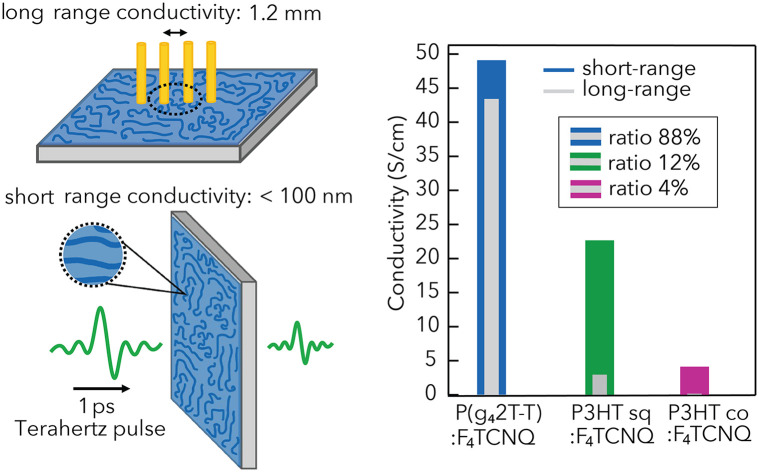

**New concepts**

The new concept here is the distance-resilience of the transport in doped conjugated polymers with high conductivity. We demonstrate that 70–90% of the conductivity is maintained over five orders of magnitude in distance (from tens of nanometers to millimeters) when the conductivity exceeds 30 S cm^−1^ in doped polythiophene films with alkyl or oligoether side chains. Typically, transport in solution-processed organic materials is dispersive and the conductivity severely drops when going from the local nanometer scale to the microscopic scale that is relevant for devices. This makes up-scaling for optoelectronic applications particularly challenging. In contrast, we find here that the local conductivity found by terahertz spectroscopy is almost the same as the one obtained over 1.2 mm by four-point probe measurements. Kinetic Monte Carlo simulations link this effect to the dielectric constant, disorder and charge confinement in the film. The concept of distance-resilience does not only have practical implications but also enhances the understanding of the transport physics in complex material systems.

## Introduction

Polythiophenes are a prominent class of conjugated polymers used as organic semiconductors in electronic and photonic applications. Advantages of such materials include their chemical tunability, flexibility and solution-processability. Recently, polythiophenes with oligoether side chains have been investigated extensively as organic mixed ionic–electronic conductors due to improved ionic transport with respect to their alkyl-bearing counterparts.^1–4^ Interestingly, the electronic conductivity is also enhanced in the oligoether derivatives when they are p-doped with molecular dopants.^5–11^ Molecular doping overcomes the intrinsically low conductivity of organic semiconductors by introducing redox-active species into the polymer structure to form radical ions *via* electron transfer.^12,13^ When the prototypical conjugated polymer poly(3-hexylthiophene) (P3HT) and its analogue with tetra-ethylene glycol side chains (P(g_4_2T-T))^1^ are doped with 2,3,5,6-tetrafluoro-tetracyanoquinodimethane (F_4_TCNQ), the conductivity is almost two orders of magnitude higher for P(g_4_2T-T).^11^

A straightforward explanation for this effect is the higher dielectric constant of the more polar P(g_4_2T-T) film, which lowers the electrostatic binding of positive charges on the polymer backbone to the negatively charged dopant counterions.^14–17^ However, other structural and energetic parameters must also be taken into account, and can vary significantly depending on the side chain nature of the polymer. For instance, it is known that F_4_TCNQ incorporates into the crystalline regions of P3HT, leading to deterioration of the polymer packing, especially if the polymer and dopant are co-processed from the same solution and aggregate before film formation.^18–20^ Alternative processing routes such as sequential doping of neat P3HT films have been proposed to reduce such doping-induced disorder.^21–25^ In contrast, coprocessing of F_4_TCNQ and P(g_4_2T-T) does not lead to aggregation and thus allows for the preparation of highly conductive films.^11^ While neat P(g_4_2T-T) forms very disordered films, the doped polymer shows improved π–π-stacking and reduced static energetic disorder.

When assessing the impact of the dielectric constant, energetic disorder and structure on the conductivity of doped polythiophene films, it is essential to consider the distance-dependence of the probed transport properties. An intrinsically high short-range mobility due to favorable local polymer conformation, packing and electrostatics will not necessarily translate to superior macroscopic transport over length-scales that are relevant to devices. Grain boundaries or energetic barriers between domains can spatially confine charges to certain regions of the film, unless connectivity is promoted over longer distances, for example by tie chains.^23,26–34^

We investigate here the parameters that impact the conductivity in the above-mentioned P3HT:F_4_TCNQ and P(g_4_2T-T):F_4_TCNQ systems over multiple length scales, in order to determine how resilient the transport is to distance. By modeling nanometer-scale measurements obtained by terahertz (THz) spectroscopy with kinetic Monte Carlo (kMC) simulations, we find that there is a moderate improvement of the short-range conductivity (by about 10 times) when co-processed P(g_4_2T-T):F_4_TCNQ is compared to P3HT:F_4_TCNQ. This is explained by a combination of local parameters such as the higher dielectric constant, lower energetic disorder and reduced charge confinement. Importantly, the difference between the two systems is amplified to a 100 times higher conductivity in P(g_4_2T-T):F_4_TCNQ, when measured across macroscopic distances by four-point-probe experiments. Indeed, only 4% of the local THz conductivity is maintained over millimeter distances for P3HT, while the polymer with oligoether side chains shows a distance–resilience (defined here as the ratio of long/short conductivity) of almost 90%.

The distance-resilience is generalized at high doping levels (achieved for alkyl side chains by sequential doping with the strong oxidant Magic Blue (MB, tris(4-bromophenyl)aminium hexachloroantimonate)). Now, even P3HT films of different regioregularities and oligoether derivatives with different side chain lengths maintain 80–90% of their nanoscale conductivity. Once this highly conductive regime is reached, the transport does not suffer anymore from structural or energetic traps that disrupt conductive pathways over longer distances, such as disorder, domain/grain boundaries or electrostatics. The generalized distance-resilience at conductivities exceeding ∼30 S cm^−1^ in doped conjugated polymers is of paramount importance for the up-scaling of organic electronic devices.

## Results and discussion

**Fig. 1:**
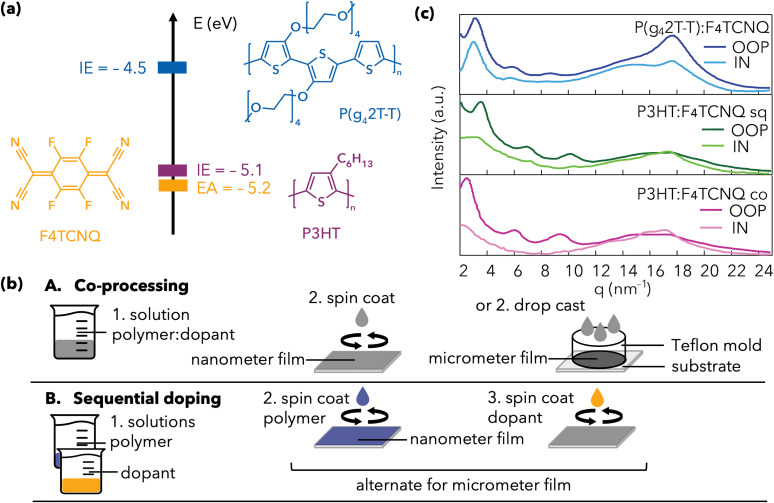
(a) Molecular structure of P3HT, P(g_4_2T-T) and F_4_TCNQ, together with the reported ionization energies for the two polymers (IE = −4.5 eV and −5.1 eV for P(g_4_2T-T) and P3HT, respectively) and the electron affinity of the dopant (EA = −5.2 eV).^11,36^ (b) Schematic for the preparation of doped polythiophene films with A. co-processing or B. sequential doping. Nanometer and micrometer thick films were prepared for the different characterization experiments. (c) Linecuts from out-of-plane (OOP) and in-plane (IN) GIWAXS data of the doped films.

The energy levels of P3HT and P(g_4_2T-T) suggest that both can be doped by F_4_TCNQ, with a higher redox offset for P(g_4_2T-T) (Fig. 1(a)). P(g_4_2T-T) was dissolved with 15 mol% F_4_TCNQ per thiophene unit in a 1 : 1 mixture of chloroform and acetonitrile and either spin coated (film thickness *d* ≈ 50 nm) or drop-cast (*d* ≈ 12 μm), as shown on the top of Fig. 1(b) and detailed in the Methods. The thick films are necessary for the THz spectroscopy. Equivalent co-processed (co) films at a similar doping level were obtained for P3HT mixed with 13 mol% F_4_TCNQ in chlorobenzene. To investigate the effect of disorder without changing the dielectric constant, an additional P3HT sample was prepared by sequential (sq) doping (bottom of Fig. 1(b)). Typically, the higher degree of order of the neat film is maintained during sequential doping of P3HT.^22,24,25,35^ The neat P3HT film was spin coated from xylene and F_4_TCNQ was deposited on top from an orthogonal solvent (dichloromethane). Micrometer thick films were obtained by alternating 10–12 polymer and dopant deposition steps.

We carried out GIWAXS experiments to determine the ordering in the doped thin films (Fig. S1–S6). Doped P(g_4_2T-T) preferentially orients edge-on to the substrate. In agreement with literature, the film depicts a clear (010) π–π-stacking peak at 17.6 nm^−1^ in the out-of-plane direction (OOP), much larger than the amorphous halo at 15 nm^−1^, as well as (100)/(200)/(300) lamellar stacking peaks at 3.0, 6.0 and 9.0 nm^−1^ (Fig. 1(c) and Table 1).^5,11^ We have previously shown that the π–π-stacking peak in undoped P(g_4_2T-T) films is negligible, so that co-processing with F_4_TCNQ enhances the backbone ordering.^11,37^ Moreover, F_4_TCNQ was found to be molecularly dispersed in P(g_4_2T-T). Given the shift of the lamellar (100) stacking peak in P(g_4_2T-T):F_4_TCNQ compared to the undoped polymer, it was concluded that the dopant incorporates in the polymer crystals and causes slightly enhanced disorder in the side chains.

**Table 1:** Peak position (*q*), peak full width at half maximum (Δ*q*), paracrystallinity (*g*) and coherence length (CCL) from the GIWAXS out-of-plane (OOP) and in-plane (IP) line cuts of the doped P(g_4_2T-T) and P3HT films

	*q* (010) (nm^−1^)	Δ*q* (010) (nm^−1^)	*g* (010) (%)	CCL (010) (nm)	*q* (100) (nm^−1^)	Δ*q* (100) (nm^−1^)	*g* (100) (%)	CCL (100) (nm)
OOP								
P(g_4_2T-T): F_4_TCNQ co	17.6	2.0	13.3	2.6	3.0	0.8	20.8	6.2
P3HT: F_4_TCNQ sq	17.7	4.8	20.7	1.1	3.4	0.7	18.9	6.7
P3HT: F_4_TCNQ co	17.7	4.1	19.1	1.2	3.4	0.7	18.3	7.0

IP								
P(g_4_2T-T): F_4_TCNQ co	17.7	2.5	15.0	2.0	2.9	0.9	22.1	5.7
P3HT: F_4_TCNQ sq	17.1	3.5	17.9	1.4	3.2	0.9	21.3	5.5
P3HT: F_4_TCNQ co	17.1	3.3	17.5	1.5	3.1	1.2	24.3	4.4

The GIWAXS patterns for the P3HT:F_4_TCNQ films indicate π–π-stacking diffractions at 17.7 nm^−1^ (OOP) and 17.1 nm (IP), without significant changes between the co-processed and sequentially doped samples (Table 1).^35^ There is a slight preference for face-on orientation, in contrast to the corresponding undoped P3HT films cast from the same solvent (Fig. S2). For the used P3HT batch (88% regio-regularity, RR), some improvement of the π–π-stacking in the IP direction occurs upon doping, especially with co-processing (see peak position, paracrystalline disorder (*g*) and coherence length of crystallites (CCL), Table S1). However, the (100) lamellar stacking peak (in the OOP direction) shifts with respect to the undoped P3HT film and shows higher paracrystallinity, indicating that F_4_TCNQ incorporates between the side chains of crystalline polymer regions. The π–π-stacking in the doped P3HT films is weaker than in P(g_4_2T-T):F_4_TCNQ, which has a much more pronounced (010) peak, higher CCL and lower cumulative disorder (in both OOP in IP directions, Fig. 1(c) and Table 1). In contrast, compared to P(g_4_2T-T):F_4_TCNQ, the lamellar ordering of the P3HT:F_4_TCNQ films is somewhat higher, which is likely related to a higher degree of disorder in the oligoether side chains.

**Fig. 2:**
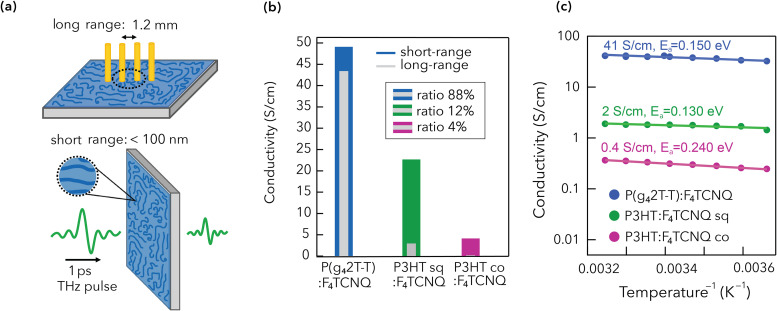
(a) Schematic of the long-range four-point probe conductivity measurement and the short-range THz spectroscopy technique. (b) Histogram of short-range conductivity in color and long-range conductivity in grey (both for thick films). The ratio between the two, which is the distance–resilience of the transport, is shown in the legend. (c) Temperature-dependent long-range conductivity of the doped polythiophene films in an Arrhenius plot: log(conductivity) *vs.* 1/temperature. The room temperature conductivity and the activation energy (*E*_a_) are shown next to the curves.

Four-point probe measurements and THz spectroscopy were used to determine the long- and short-range conductivity of the doped polythiophene films, respectively (Fig. 2(a)). The distance probed for the long-range transport is defined by the electrode spacing of 1.2 mm in the four-point probe experiment. In THz spectroscopy, the charges are displaced by the electric field of a short THz pulse. The probed distance is determined by the duration of the THz pulse of around one picosecond, during which the charges move several tens of nanometers. The short-range conductivity (*σ*_short_) can be interpreted as the intrinsic limit of conductivity in the doped polythiophene films, while the long-range conductivity (*σ*_long_) can be interpreted as a resilience over distances that are relevant for macroscopic device geometries. We define their ratio (*σ*_long_/*σ*_short_) as the distance–resilience of the transport. Here, *σ*_long_ is clearly superior for P(g_4_2T-T):F_4_TCNQ (43 S cm^−1^), followed by sequentially doped P3HT (3 S cm^−1^) and finally the co-processed P3HT sample (0.2 S cm^−1^, Fig. 2(b)). Although the trend is similar, *σ*_short_ is only improved by about 10 times between the co-processed doped P3HT and P(g_4_2T-T) films (compared to a >100 times improvement for *σ*_long_). The film thickness can be ruled out as the origin of this effect, as both measurements were recorded using micrometer films. We also verified that a similar *σ*_long_ was found for the micrometer- and nanometer-thick samples, ruling out any loss of conductivity due to the thick sample preparation or film roughness (Table S2 and Fig. S7). Instead, the effect is caused by a fundamentally different distance–resilience in the conductivity of the two polymers. The doped P(g_4_2T-T) film maintains 88% of the intrinsic THz conductivity over a distance of 1.2 mm. In contrast, doped P3HT has only few percent of the conductivity remaining over longer distances, with a small improvement with sequential doping from 4% (co) to 12% (sq).

**Fig. 3:**
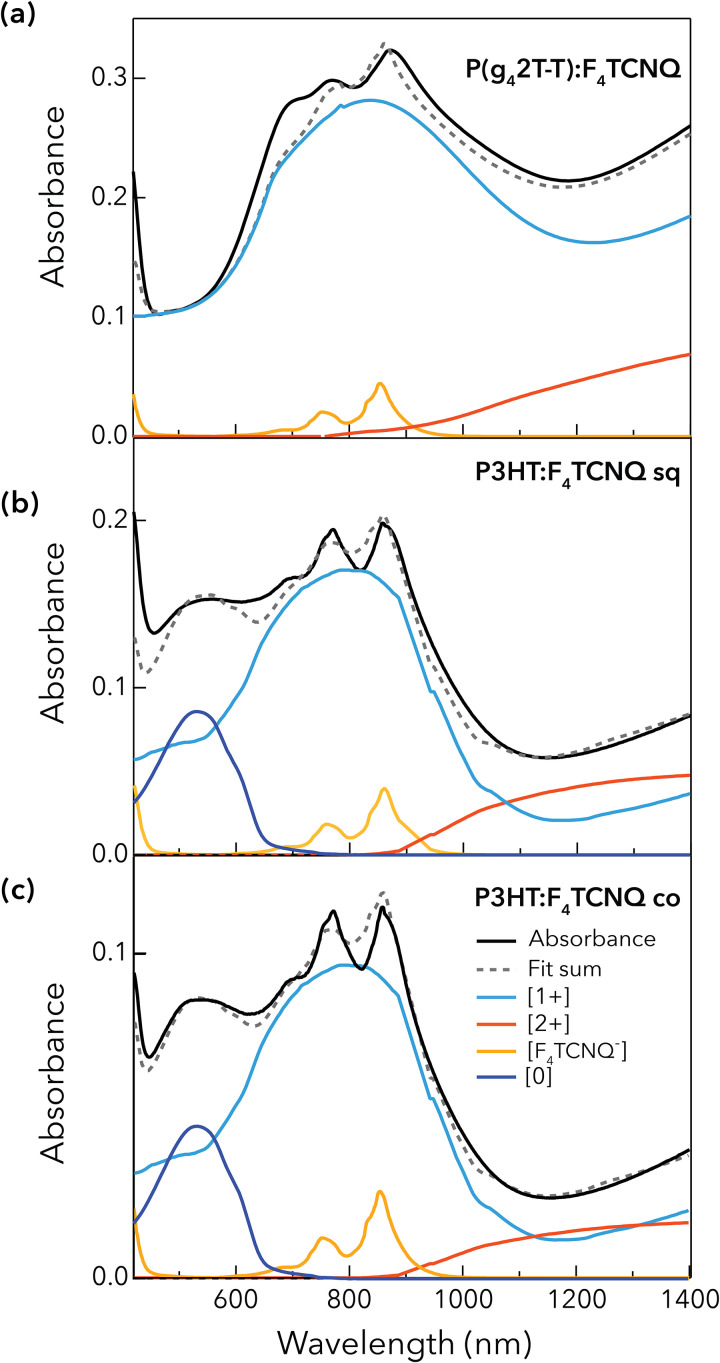
Steady-state absorbance spectra of the three investigated doped thin films: (a) P(g_4_2T-T):F_4_TCNQ, (b) P3HT:F_4_TCNQ (sq) and (c) P3HT:F_4_TCNQ (co). The experimental spectra (black lines) were fit to the sum (dashed line) of the scaled individual spectral components (colored lines), as extracted from spectroelectrochemistry or obtained from the literature (see main text).

To elucidate if a different transport regime affects the two polymers, we have measured *σ*_long_ of the doped films as a function of temperature. We find that the conductivity always increases at higher temperatures, which is typical for thermally activated hopping transport. The activation energy (*E*_a_) to charge transport is extracted from an Arrhenius plot (Fig. 2(c)). A comparable *E*_a_ of 130–150 meV is found for P(g_4_2T-T):F_4_TCNQ and P3HT:F_4_TCNQ (sq), while the higher *E*_a_ in P3HT:F_4_TCNQ (co) points to a less ordered nanostructure of this film. Charge transport and activation energy depend on the doping level.^38–42^ Here, the Arrhenius plot indicates that all the investigated films are in a similar doping regime and are ruled by similar transport physics. To confirm the similar doping level, we turn to the steady-state absorbance spectra of the three thin films, shown in Fig. 3. They are decomposed into different spectral components. The neutral polymer sites (denoted as [0]) absorb in the 400–650 nm region. When doped, the polymer segments can be oxidized once [1+] or twice [2+], yielding characteristic spectral signatures in the near-infrared (NIR) region.^43–46^ We have determined the absorption cross section spectra of the different oxidized species using a combination of spectroelectrochemistry and chronoamperometry, following our previously reported procedure (Fig. S8 and S9).^47^

In addition to the polymer signatures, the ionized F_4_TCNQ^−^ peaks are present at 760 and 860 nm.^48^ As their cross sections in solution are known, we deduce the charge densities present in the doped films, given that each singly ionized dopant generates one positive charge on the polymer backbone.^6^ This yields a lower bound estimate, under the assumptions that the absorbance cross section of F_4_TCNQ^−^ is the same in solution and film,^35^ and that each F_4_TCNQ molecule is only ionized once (neglecting double doping).^5,6^ The resulting charge density (including bound and mobile charges) is similar for the doped P(g_4_2T-T) and P3HT samples (2–4 × 10^20^ cm^−3^, Table S3). Alternatively, we determined the total charge density ([Charge]_tot_ = [1+] + 2 × [2+]) from the absorbance and cross sections of the polymer charged species and find only slightly higher values of 5–7 × 10^20^ cm^−3^ (Table 2). Charge densities over 10^20^ cm^−3^ seem high, but agree with reports on doped polymers probed by a variety of techniques.^49–52^ Independently of the absolute value, the important point is that the charge density and the transport regime are similar for the P(g_4_2T-T) and P3HT samples and are thus not responsible for the observed differences in conductivity.

**Table 2:** Conductivity and charge densities in doped polythiophene films: long-range conductivity from four-point probe measurements (*σ*_long_), short-range conductivity from THz spectroscopy (*σ*_short_), distance–resilience of the conductivity (*σ*_long_/*σ*_short_), total polymer charge density ([Charge]_tot_ = [1+] + 2[2+]), relative densities of the different oxidized species ([0], [1+] and [2+]) and ratio of the doubly to singly charged species ([2+] : [1+])

Polymer	Dopant	*σ*_long_ (S cm^−1^)	*σ*_short_ (S cm^−1^)	*σ*_long_/*σ*_short_ (%)	[Charge]_tot_ (10^20^ cm^−3^)	[0] %	[1+] %	[2+] %	[2+] : [1+]
P(g_4_2T-T)	F_4_TCNQ co	43^b^	49^b^	88	7.3	0	80	20	0.3 : 1
	F_4_TCNQ im	32	40	80	3.3	0	82	18	0.2 : 1
	MB im	20	26	77	4.2	0	39	61	1.5 : 1
P3HT^a^ 88% RR	F_4_TCNQ co	0.2^b^	4^b^	5	4.8	22	68	11	0.2 : 1
	F_4_TCNQ sq	2.8^b^	23^b^	13	5.9	21	63	16	0.3 : 1
	MB im	60	67	90	7.5	8	53	39	0.8 : 1
P3HT 98% RR	F_4_TCNQ co^c^	3.9	29^b^	13	9.7	28	59	13	0.2 : 1
	F_4_TCNQ sq	1.8	10^b^	18	5.3	22	68	10	0.1 : 1
	F_4_TCNQ im	5.9	11	54	2.4	38	59	3	0.1 : 1
	MB im	108	122	89	6.7	8	57	35	0.6 : 1
P(g_3_2T-T)	F_4_TCNQ im	330	395	84	9.4	0	68	32	0.5 : 1
	MB im	140	210	67	13	0	45	55	1.2 : 1

a Batch used throughout the text, unless stated otherwise.

b Conductivities measured on micrometer thick films. All other data in the table is shown for nanometer thicknesses indicated in Table S3. Differences between *σ*_long_ for nanometer and micrometer films were negligible (Table S2). For the more conductive films, the same film could be used for optical, THz and four-point probe measurements.

c Due to film inhomogeneity, absolute values in this sample might be exaggerated, but trends and ratios are correct.

Another parameter that impacts the conductivity of doped samples is the co-existence of different oxidized species in the polythiophene films. The singly charged species (referred to as polarons) and doubly charged species (referred to as polaron pairs or bipolarons depending on their spin characteristics) have distinct transport properties. For polythiophenes, the conductivity first increases and then stabilizes or drops with increasing [2+] concentration.^43,53,54^ It is therefore essential to determine the ratio of the different oxidized species in our P3HT:F_4_TCNQ and P(g_4_2T-T):F_4_TCNQ samples (Table 2). The first observation is that the P(g_4_2T-T):F_4_TCNQ film shows no signature of the neutral polymer, while about 20% of neutral sites remain in the ordered and disordered regions of the P3HT:F_4_TCNQ samples. The incomplete doping can be related to the small energetic offset between P3HT and the dopant (Fig. 1(a)). In contrast, the large offset with P(g_4_2T-T) allows this polymer to be completely doped. The second observation is that the [2+] concentration is always significantly lower than that of [1+], as highlighted by the ratio of the two charged species, which is around 0.2 : 1. In this moderate doping regime, we have previously shown that the [2+] species are located in the more amorphous parts of the film and have a beneficial effect on the transport.^43,47^ However, the small trends between the three samples are not significant within the experimental error and film-to-film variation (Fig. S10 and Table S3), so that the [2+] : [1+] ratio is unlikely to be the primary cause of the different conductivity behaviour.

**Fig. 4:**
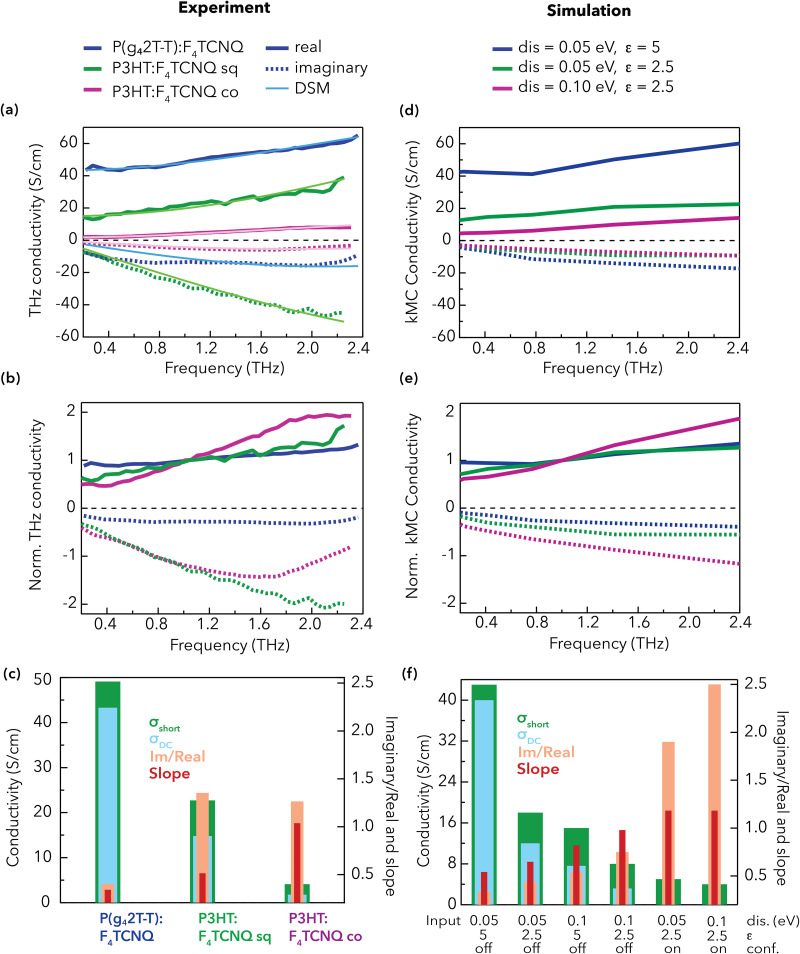
(a) Complex conductivity spectra of the doped polythiophene films obtained from THz spectroscopy (real part as solid lines, imaginary part as dashed lines) and fits with the Drude–Smith model (DSM, thin lines). (b) Experimental complex conductivity spectra normalized by the magnitude of the real part at 1 THz. (c) Selected observables visualized in different histograms: real conductivity at 1 THz (*σ*_short_), zero frequency conductivity extrapolated from the DSM (*σ*_DC_), ratio between the imaginary and real part at 1 THz (Im/Real), and slope of the real part between 0.8 and 1.4 THz (Slope). (d) Complex conductivity spectra simulated by kMC simulations for different dielectric constants and static energetic disorder (scaled by a factor 20 to match the experiment). (e) Simulated complex conductivity spectra normalized in the real part at 1 THz. (f) Same output parameters as in (c) obtained from kMC simulations under different conditions (disorder, dielectric constant and confinement).

Knowing that the number and nature of doping-induced charges are comparable, we hypothesize that the charge mobility plays the major role in differentiating the P(g_4_2T-T):F_4_TCNQ and P3HT:F_4_TCNQ samples. This mobility represents an average value, as not all charges contribute equally to the transport. Indeed, it has been shown that only a small fraction of the charges in doped P3HT films actively participate to the conductivity at a given point of time.^13,43^ For instance, charges in amorphous film regions or those that are bound to the ionized dopant are expected to contribute less. We turn to the details of the THz data to selectively investigate the nanoscale mobility of the charges that contribute to the transport. Their transport characteristics are reflected by the shape of the complex conductivity spectra (Fig. 4(a)). We draw attention to three observables, namely (i) the amplitude of the real part, (ii) the relative amplitude of the imaginary with respect to the real part and (iii) the slope of the real part (Fig. 4(b and c) and Fig. S11). The amplitude of the real part at 1 THz reflects the short-range conductivity (Fig. 2(b)). We found a 10-fold increase in the short-range conductivity when going from co-processed P3HT:F_4_TCNQ to P(g_4_2T-T):F_4_TCNQ. To highlight the other two effects, we normalized the real conductivity at 1 THz and scaled the imaginary part by the same factor (Fig. 4(b)). The slope of the real part becomes flatter when going from P3HT:F_4_TCNQ (co), *via* P3HT:F_4_TCNQ (sq) to P(g_4_2T-T):F_4_TCNQ. Also, the two P3HT samples have a similar imaginary-to-real ratio, but their negative imaginary part is much more pronounced than for doped P(g_4_2T-T).

The complex conductivity spectra of organic materials are commonly interpreted using the Drude–Smith model (DSM), which allows to disentangle the charge mobility from their density. Being derived for band-like transport in the presence of backscattering or a restoring force, its applicability in hopping systems is not *per se* given.^55^ For polymers, ballistic transport can phenomenologically be thought of as delocalized transport along conjugated segments, while the hopping can be associated with charges being bound by one or more of a multitude of effects, including structural and energetic disorder, coupling to vibrational modes, Coulomb attraction to the ionized dopant or confinement in an crystalline aggregate.^56^ In the framework of the DSM (eqn (1)), the negative imaginary part in all our doped samples confirms that hopping dominates the short-range transport (similar to the thermally activated long-range transport, Fig. 2(c)). Moreover, the flatter real part and smaller relative imaginary part in P(g_4_2T-T):F_4_TCNQ indicate that the charges in this sample are less bound.

Quantitatively, eqn (1) is fitted to the complex conductivity spectra (Fig. 4(a) and Table 3):1
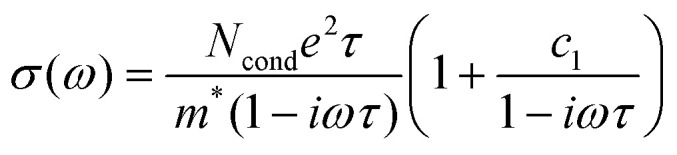


**Table 3:** Fit parameters from the DSM analysis: density of charges that participate in transport (*N*_cond_), percentage of total charges contributing to transport (*N*_cond_/[Charge]_tot_), scattering time *τ*, localization parameter *c*_1_, effective mobility (*μ*_eff_) and average mobility (*μ*_av_). The values in parentheses indicate the range in which an acceptable fit can be obtained, as the DSM allows for some flexibility (Fig. S12). Additionally, we calculated the zero-frequency conductivity from the extrapolated DSM fits (*σ*_DC_)

	P(g_4_2T-T):F_4_TCNQ	P3HT:F_4_TCNQ sq	P3HT:F_4_TCNQ co
*N*_cond_ (10^19^ cm^−3^)	4 (3–8)	10 (5–15)	0.4 (0.3–0.7)
*N*_cond_/[Charge]_tot_	5% (4–11%)	17% (8–25%)	0.8% (0.6–1.5%)
*τ* (fs)	25 (15–31)	17 (15–24)	35 (27–45)
*c*_1_	−0.74 (0.73–0.77)	−0.95 (0.95)	−0.93 (0.92–0.97)
*μ*_eff_ (cm^2^ V^−1^ s^−1^)	6.7 (3.6–8.7)	0.9 (0.8–1.2)	2.5 (1.4–2.5)
*μ*_av_ (cm^2^ V^−1^ s^−1^)	0.37 (0.36–0.40)	0.15 (0.11–0.20)	0.02 (0.01–0.03)
*σ*_DC_ (S cm^−1^)	43	15	2

Here, *σ*(*ω*) is the frequency-dependent complex conductivity, *N*_cond_ is the density of conductive charges, *τ* is the scattering time, m* is the effective mass (≈1.7),^57^*ω* is the frequency, and *c*_1_ is the localization parameter (−1 ≤ *c*_1_ ≤ 0). Interestingly, *N*_cond_ is always below the total density of charges from the absorbance spectra ([Charge]_tot_, Table 2). For P(g_4_2T-T):F_4_TCNQ and P3HT:F_4_TCNQ (sq), less than 5–20% of the total charges contribute to the transport (Table 3). This drops to only 1% in the case of P3HT:F_4_TCNQ (co), partially explaining the low short-range conductivity of this film. Moreover, in both P3HT samples, the localization parameter approaching −1 is indicative of strongly bound charges, while doped P(g_4_2T-T) shows a smaller localization around −0.74. The scattering time (representative only in the context of ballistic transport) has no trend between samples. From *τ* and *c*_1_ we calculate an effective short-range mobility (*μ*_eff_) according to eqn (2):2
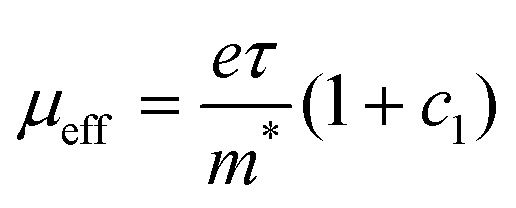


We find a high *μ*_eff_ of 6.7 cm^2^ V^−1^ s^−1^ for the charges that contribute to transport in P(g_4_2T-T):F_4_TCNQ films (Table 3). Both P3HT:F_4_TCNQ films have a lower short-range mobility of 1–2 cm^2^ V^−1^ s^−1^, but the fraction of charges participating in the transport is higher in the sequentially doped film, leading to an overall higher short-range conductivity. To take this effect into account, we averaged the short-range mobility over all conductive and non-conductive charges in eqn (3):3
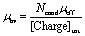


The obtained average mobilities closely follow the trend in short-range conductivity of the three doped samples and explain the differences in the conductivity at similar doping level (Table 3). Finally, we evaluate if the DSM can account for the distance-dependence of the transport, by extrapolating the DSM fit to zero frequency (Fig. S11) to find the DC conductivity (*σ*_DC_, Fig. 4(c) and Table 3). For P(g_4_2T-T):F_4_TCNQ, this predicts a distance–resilience of 88%, which perfectly matches the experimental ratio (Fig. 2(b)). However, for the doped P3HT films, the extrapolated DC conductivity is overestimated by 5–10 times.

To overcome the lack of physical meaning behind the phenomenological DSM parameters, we have investigated the short-range transport using kMC simulations. Specifically, we target the effect of the energetic disorder and dielectric constant on hopping (Fig. S13). Hopping-type transport is justified here by the thermally activated conductivity (Fig. 2(c)) and the negative imaginary part of the THz spectra (Fig. 4(a)). As detailed in the Methods, the kMC simulations were run using a rectangular grid of 10 × 10 × 10 with a lattice parameter of 1.8 nm and allowing for only nearest-neighbour hopping events (input parameters in Table S4).^33,58^ A constant dopant concentration of 10% compared to the total number of sites was used to mimic the experimental conditions (both charges and dopant ions were randomly added on the grid). We have included the energetic disorder in the density of states (DOS) of the hopping sites. Note that this is a short-range parameter (representative of the local polymer ordering) and therefore differs from the long-range paracrystalline disorder obtained by GIWAXS. The Coulomb interactions of charges with all surrounding dopant ions and other charges were recalculated after every hopping event at the chosen dielectric constant.

There is overall good agreement between the simulated complex conductivity spectra and the experimental data (Fig. 4 and Fig. S14). Decreasing the energetic disorder from 0.1 to 0.05 eV is sufficient to reproduce the increase and flattening in the real conductivity when going from co-processed to sequentially doped P3HT:F_4_TCNQ. Thus, a higher degree of nanoscale ordering of the polymer chains is the main reason for the improved short-range conductivity of P3HT:F_4_TCNQ (sq). In addition to lower disorder, a higher dielectric constant (*ε* = 5 instead of 2.5) is necessary to simulate the further increase in the real conductivity of P(g_4_2T-T):F_4_TCNQ, in line with the experimental increase in polarity from *ε* = 2.7 to 4.4.^30^ Binding of charges to the ionized dopant reduces the mobility of doped polymers, especially in the low to moderate doping regime.^13,30,59^ At high doping levels, the electrostatic potential wells of the dopants overlap and bound charges become mobile.^27,60,61^ As such, a higher dielectric constant can mitigate Coulomb trapping in organic semiconductors, which can be thought of as reducing the restoring force in the Drude–Smith model.^14,16,17,62,63^ Our results demonstrate that Coulomb effects are indeed important at the doping level investigated here, and that the use of oligoether side chains is an effective chemical design strategy to overcome this limitation.

The simulation with reduced disorder and high dielectric constant accounts for all features of the experimental P(g_4_2T-T):F_4_TCNQ spectrum, including the real slope and imaginary-to-real ratio (Fig. 4(f)). The significant DC conductivity from the kMC simulation at zero frequency also predicts the high distance–resilience of transport in these films (Table S5). However, for both doped P3HT films, the simulations (with varied disorder and dielectric constant) underestimate the relative imaginary part and overestimate the DC conductivity (Fig. 4). Therefore, there must be an additional parameter that causes the charges in P3HT to be more bound and less mobile. We suggest that this is due to spatial confinement of the charges in the film, meaning that certain regions are inaccessible to mobile charges. The effect is somewhat related to the high π–π stacking paracrystallinity of both P3HT samples (Table 1), but encompasses more complex phenomena such as grain boundaries, energetic barriers between crystalline and amorphous domains, undoped regions (20% neutral polymer segments are left), dopant aggregates or lacking tie chains for connectivity.^18,23,26–34,64^ To test our hypothesis, we simulated the confinement by removing one of the periodic boundary conditions in the kMC simulations, such that a charge is confined to 10 sites in the (*z*-)direction of the applied AC electric field (Fig. S14 and Fig. 4(f)). This severe confinement overshoots the experimentally observed effects, but leads to the right trends: an increase of the negative imaginary part, a steeper slope of the real part and a suppression of the DC conductivity. We conclude that the spatial confinement is indeed an additional parameter that governs the transport and its distance–resilience in doped polythiophenes together with the dielectric constant and energetic disorder.

Finally, we generalized our experimental findings to additional thiophene-based polymers and a stronger dopant, namely a P3HT batch with higher regioregularity (98% *versus* 88%), a derivative of P(g_4_2T-T) with a shorter oligoether side chain (P(g_3_2T-T))^5^ and Magic Blue (MB) dopant (Fig. S15–S18). To reach high conductivities with P3HT, an optimized sequential doping protocol called immerse doping (im) was used, whereby the undoped polymer film was immersed in a solution of the dopant (see Methods). We find that increasing the regioregularity of P3HT typically leads to higher conductivity for a given doping condition, but has a marginal effect on the distance–resilience and shape of the THz spectrum (Table 2 and Fig. S15). Importantly, the P3HT:MB (im) films with 88% and 98% regioregularity both show high conductivity (*σ*_long_ = 60 and 108 S cm^−1^, respectively), improved depletion of the neutral sites (only 8% left), a higher [2+]/[1+] ratio and a distance–resilience of 90%. This is related to the increased oxidation strength of the dopant and to preferential insertion of MB into amorphous polymer regions, limiting doping-induced disordering of the crystalline regions.^65,66^

However, MB is not the optimal dopant for the polymers with oligoether side chains (P(g_4_2T-T) and P(g_3_2T-T)), which both show reduced conductivity and lower distance–resilience when doped with MB (im) compared to F_4_TCNQ (im) (Table 2). This is due to an overdoping effect, where a too high [2+] density, exceeding that of [1+], limits the conductivity.^43,53,54^ The best conductivity (*σ*_long_ = 330 S cm^−1^, 84% distance–resilience) is achieved with P(g_3_2T-T):F_4_TCNQ (im), ten times higher than in the equivalent P(g_4_2T-T) film. For P(g_4_2T-T), immerse doping is less successful than co-processing owing to the very disordered nature of the undoped polymer film.^11^ Better ordering of the shorter side chains improves the lamellar stacking in undoped P(g_3_2T-T).^5^ It was shown that F_4_TCNQ does not disrupt this ordering and further improves the π–π stacking, explaining the excellent performance of the P(g_3_2T-T):F_4_TCNQ system.^5^ Overall, we find that the absolute conductivity is highly dependent on the nature, processing conditions and film structure of each polymer : dopant pair. Nevertheless, the concept of distance–resilience depends much less on those parameters. In fact, we show that all films exceeding a *σ*_long_ of ∼20–30 S cm^−1^ have an important resilience of their conductivity over several orders of magnitude in distance. Once this high-conductivity regime is reached, the transport does not suffer anymore from structural or energetic traps that disrupt conductive pathways over longer distances, such as disorder, domain/grain boundaries or electrostatics.

## Conclusion

In conclusion, we explore here the distance–resilience of the transport in polythiohene : dopant systems. We start with a detailed investigation of F_4_TCNQ-doped films in the moderate doping regime and show superior conductivity of a polythiophene derivative with oligoether side chains (P(g_4_2T-T)) with respect to P3HT. For co-processed films, the long-range conductivity is enhanced by two orders of magnitude in P(g_4_2T-T):F_4_TCNQ compared to P3HT:F_4_TCNQ. Importantly, doped P(g_4_2T-T) retains 90% of its conductivity from the nanometer to millimeter scale, in contrast to just a few percent distance–resilience (defined as the ratio of long-range to short-range conductivity) for doped P3HT. All investigated samples have a charge density ≈5.0 × 10^20^ cm^−3^ and a low concentration of doubly charged species ([2+] : [1+] ≈ 0.2 : 1). The increase in conductivity with P(g_4_2T-T) is related to changes in the average mobility, meaning that either more charges contribute to the transport or that the conductive charges are more mobile. This is confirmed by a Drude–Smith analysis of the THz conductivity data, which shows that the average nanoscale mobility scales with the short-range conductivity (P(g_4_2T-T):F_4_TCNQ > P3HT:F_4_TCNQ (sq) > P3HT:F_4_TCNQ (co)). Within this framework, the more negative imaginary part of the complex conductivity also points to more bound charges in the doped P3HT:F_4_TCNQ films.

We carried out kMC simulations to pinpoint this effect to physically meaningful parameters. We find that decreasing the energetic disorder (representative of local polymer ordering) from 0.1 to 0.05 eV is sufficient to reproduce the increase in short-range conductivity when going from co-processed to sequentially doped P3HT:F_4_TCNQ. However, this simulation underestimates the relative imaginary part and overestimates the DC conductivity at zero frequency for both doped P3HT samples. Additional spatial confinement of the transport is necessary to capture these phenomena in the simulations. This agrees with the high paracrystalline disorder in the doped P3HT films and with the incomplete doping (20% neutral sites remain that might not accommodate charges for transport). Grain boundaries, energetic barriers between amorphous and crystalline domains, dopant aggregates or lacking tie chains are further limitations to long-range transport. In contrast, kMC simulations with reduced energetic disorder (0.05 eV) and a high dielectric constant (*ε* = 5), without need for any additional confinement, account for all features of the complex conductivity of P(g_4_2T-T):F_4_TCNQ films, including the high short-range conductivity, reduced relative imaginary part and high distance–resilience of the transport. Our results thus demonstrate that Coulomb effects are important at the moderate doping level investigated here, and that the use of oligoether side chains is an effective chemical design strategy to overcome this limitation. Moreover, we show that the improved processability and polymer ordering over multiple length scales is essential to foster the nanoscale conductivity of P(g_4_2T-T):F_4_TCNQ, and to maintain 90% of its high conductivity up to millimeter distances, thus over five orders of magnitude.

We then extend the concept of distance-resilience to a larger set of polymer and dopant materials in the high doping regime. We find that the absolute conductivity strongly depends on the nature, processing conditions and film structure of each polymer : dopant pair, but the distance-resilience is quite independent of those parameters. Generally, all films exceeding a conductivity of ∼30 S cm^−1^ show highly distant-resilient transport. We thus demonstrate that a high-conductivity regime can be reached in molecularly doped conjugated polymers, where structural, energetic and electrostatic traps play a minor role in limiting the conductivity over macroscopic distances. This finding is highly relevant for the upscaling of organic electronic devices.

## Conflicts of interest

There are no conflicts to declare.

## Supplementary Material

MH-012-D5MH00620A-s001

## Data Availability

The data that support the findings of this study are available as open access in the BORIS Repository of the University of Bern at https://boris-portal.unibe.ch/handle/20.500.12422/217215 Supplementary information: Experimental methods, details on the simulations and additional figures and tables as mentioned in the main text. See DOI: https://doi.org/10.1039/d5mh00620a
