# Supplementary material for: Distance–resilient conductivity in p-doped polythiophenes
Source: Mater Horiz. 2025 Aug 25;12(24):10827–38. doi: 10.1039/d5mh00620a (PMC12439127; doi:10.1039/d5mh00620a)
Supplement: MH-012-D5MH00620A-s001 [file MH-012-D5MH00620A-s001.pdf]

Supplementary Information (SI) for Materials Horizons.
This journal is © The Royal Society of Chemistry 2025

Supplementary Information

Distance-resilient conductivity in p-doped polythiophenes

Eva Röck[1], Demetra Tsokkou[1], Basil Hunger[1], Maximilian M. Horn[1], Sepideh Zokaei[2], Renee Kroon[2,3], Jesika Asatryan[4], Jaime Martín[4], Christian Müller[2]*, Martijn Kemerink[5]* and Natalie Banerji[1]*

[1] Department for Chemistry, Biochemistry and Pharmaceutical Sciences, University of Bern, Freiestrasse 3, 3012 Switzerland.

[2] Department of Chemistry and Chemical Engineering, Chalmers University of Technology, 412 96 Göteborg, Sweden.

[3] Laboratory of Organic Electronics, Linköping University, SE-581 83 Linköping, Sweden.

[4] Instituto de Ciencia y Tecnología de Polímeros, CSIC, Juan de la Cierva 3, 28006 Madrid, Spain.

[5] Institute for Molecular Systems Engineering and Advanced Materials, Heidelberg University, Im Neuenheimer Feld 225, 69120 Heidelberg, Germany.

Methods

Materials

The synthetic route of P($g_4$2T-T) was described previously.[1] The number-average molecular weight is M_n = 24 kg/mol and the polydispersity index PDI = 3.3. P($g_3$2T-T) was purchased from 1-Material. P3HT was purchased from Solaris Chem with a regioregularity of 88%, M_n = 30-50 kg/mol and PDI = 2.4, or from Ossila with a regioregularity of 98%, M_n = 29 kg/mol and PDI = 2.1. F_4TCNQ was purchased from Ossila and Magic Blue (MB, tris(4-bromophenyl)aminium hexachloroantimonate) from Sigma Aldrich.

Film preparation

Neat films of P($g_4$2T-T) were spin-coated from a 20 g/l solution in chloroform and used for spectroelectrochemistry and for immerse doping (immersion time 60-120 seconds in a 1.5 g/l solution of F_4TCNQ or a 0.5 g/l solution of MB in acetonitrile). The same films (thicknesses in Table S2) could be used for optical, conductivity and THz measurements. Alternatively, 5 g/l p($g_4$2T-T) was co-processed with 15 mol% F_4TCNQ per thiophene unit in a 1:1 mixture of chloroform and acetonitrile. The polymer and dopant were first dissolved separately for 2 hours at room temperature, then mixed for 1 hour before spin-coating nanometer films (for optical characterization) and drop-casting micrometer thick films (for THz spectroscopy) in a Teflon mold. For co-processed films, 10 g/l P3HT was mixed with 13 mol% F_4TCNQ per thiophene unit in chlorobenzene in an analogue manner. Again, we prepared both spin-coated thin films (nanometer thickness) and drop-casted thick films (micrometer thickness, stirring and drying at 80°C to avoid aggregation). For sequential doping, P3HT films were prepared from a 10 g/l solution in xylene and sequentially doped by spin-coating an orthogonal doping solution of F_4TCNQ (1g/l in dichloromethane) on top. This technique allowed to produce thin films of ~30 nm and thick films by alternating spin-coating of pristine P3HT and F_4TCNQ (~2 µm) for 10-12 times. The alternating sequential doping is based on the additive solution deposition.[2] The long-range conductivity was controlled and did not vary substantially up to 12 layers. For immerse doping with MB, neat P3HT films were spin-coated from a 15 g/l dichlorobenzene solution (stirred at 80 C for 2 hours), spin coated at 1000 rpm and immersed in a 0.5 g/ml MB solution (5 minutes). Thicker films were used when doped with F_4TCNQ. Hear, neat P3HT was spin coated from a 40 g/l dichlorobenzene solution, spin-coated at 700 rpm and then immersed in a 1.5 g/l solution of F_4TCNQ (1 hour). The same films were used for all measurements. P($g_3$2T-T) was dissolved in chloroform (5 g/l) and stirred at 45°C overnight, then the

temperature was elevated to 60°C for 30 min and then returned to 45°C before spin-coating at 750 rpm. The undoped film was used for spectroelectrochemistry or chemically doped by immersion for 60 seconds in 1.5 g/l of F4TCNQ or 0.5 g/l of MB in acetonitrile.

Steady-state absorbance and spectroelectrochemistry

The electronic transitions for each thin film were recorded with an absorbance spectrometer equipped with an integration sphere (Perkin Elmer Lambda 950). For spectroelectrochemistry, the neat polymer films were spincoated onto ITO-coated quartz substrates. A film area of 0.3 x 0.4 mm was delimited with Capton tape and exposed to the electrolyte (degassed 100 mM $TBAPF_6$ in acetonitrile) in a solvent-resistant cell containing an AgCl counter-electrode. A PalmSens4 bipotentiostat was used to apply voltage steps of 0.1 over a range of + 0.4 V to -1.2 V while recording current transients. In parallel, spectral changes were recorded with two detectors from Ocean Optics (visible and near-infrared from 400 nm to 1600 nm). For the light source, a HL 2000 lamp (Ocean Optics) was used.

White light interferometry (WLI)

Thickness and roughness of the prepared samples were determined using an optical profiler with 5x magnification (Bruker CountourGT). The thickness was calculated as the average from at least 12 positions close to the center of the polymer film.

Grazing-incidence wide-angle scattering (GIWAXS)

The GIWAXS measurements were performed at the BL11 NCD-SWEET beamline at ALBA Synchrotron Radiation Facility (Spain). The scattering patterns were recorded using a Rayonix® LX255-HS area detector, which consists of a pixel array of 1920 × 5760 pixels (H × V) with a pixel size of 44 × 44 μm^2. The incident X-ray beam energy was set to 12.4 eV (0.9998 Å) using a channel cut Si (1 1 1) monochromator. The incident angles were set to 0.05, 0.1, 0.12, 0.15°. Data are expressed as a function of the scattering vector (q), which was calibrated using Cr_2O_3, obtaining a sample to detector distance of 201.7 mm and 200.5 mm for two consecutive beam times. To perform the analysis of the diffraction patterns, the 2D patterns were integrated, corrected with respect to the reference and the 1D profiles were extracted using the software Fit2D. The coherence length (CCL) of the ordered domains in the films is roughly estimated by the Scherrer equation:

$$CCL = \frac{2\pi K}{\Delta q}$$

where K is the shape factor (~0.9-1) and Δq is the full width at half maximum of the peak.

The cumulative disorder was calculated by the paracrystallinity parameter g:

$$g = \sqrt{\frac{\Delta q}{2\pi q_0}}$$

where q_0 is the peak center position.

Long-range conductivity

The long-range conductivity of the doped polymer films was measured using a compact four-point probe system (Ossila Four Point Probe TypeG) at room temperature (1.2 mm distance between contacts). The room temperature values are dimension- and thickness- corrected results repeated on three spots on the same thick films as used for THz spectroscopy. The conductivity was also measured for several thin films, used for the optical spectroscopic characterization. Additionally, the conductivity was measured at 25 different temperatures between 40°C and -190°C using a Linkam LTS420 thermal stage. The sample was placed on the liquid nitrogen cooled thermal stage with four spring-loaded tungsten electrodes to probe the electrical performance in a linear four-point probe geometry.

THz time domain spectroscopy (THz-TDS)

The THz time domain spectroscopy (THz-TDS) setup is based on the output of a regeneratively-amplified Ti:sapphire laser system (Coherent Astrella). The 800 nm output with an average power of 6.5 W and ~35 fs pulse duration at 1 kHz repetition rate was reduced to 35 μW to generate the THz beam. The THz pulses were generated via optical rectification in a 2 mm [110] ZnTe crystal. The transmitted THz pulse was detected by electro-optic sampling in another 1 mm [110] ZnTe crystal. The ZnTe generation crystal, sample holder and the detector were kept in nitrogen atmosphere to exclude THz absorption by air humidity. The response of the THz electric field to the charges in the doped films yields the ground state conductivity. It was obtained from the Fourier transform of the THz response to the doped film and the Fourier transform of the transmitted THz pulses through the substrate.

Kinetic Monte Carlo simulations

Kinetic Monte Carlo (kMC) simulations describe the motion of charge carriers in a doped disordered system. kMC simulations similar to the ones presented in previous studies [3-4] were performed for a dopant concentration of 10% compared to the total number of sites , which is

comparable to the one used experimentally, on a rectangular grid of 10x10x10 with a lattice parameter of 1.8 nm. In each simulation the results were averaged over at least 10 random configurations. Typically, simulations ran for long times to assure steady state conditions and that charge carriers reached room temperature. In these simulations, the dielectric constant and the energetic disorder were varied. The disorder was set to a generic value of 50-100 meV based on the typical range previously reported for simulations and experiments.[5-8] Upon initialization, both charges and dopant anions were randomly added on the lattice sites at equal concentration. All Coulomb interactions of mobile charges with all surrounding dopants and all other charges were recalculated after every hopping event. The dopants were included as static charges on the lattice. We considered only nearest-neighbour hopping events. The initial energy level of charge carriers was chosen from a Gaussian density of states and hopping rates were calculated using the Miller Abrahams expression. The energy barrier for charges to occupy a dopant site was taken as the energy difference between the HOMO of the polymer and the LUMO of the dopant ($\Delta E = 0.24$ eV). The energetic disorder parameter was included in the energy distribution of the sites. All obtained conductivities were scaled by a factor 20 to match the experimental data.

S2. GIWAXS

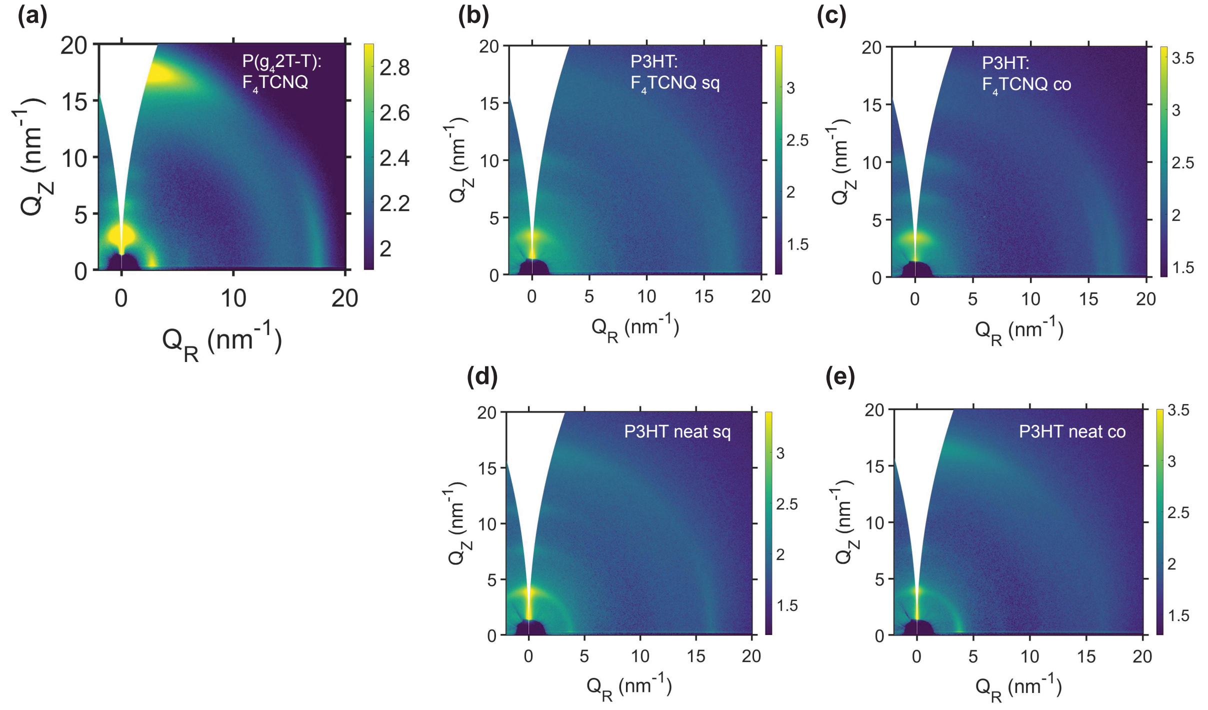

Figure S1. GIWAXS 2D patterns corresponding to the linescans shown in the main text for doped polythiophene thin films (a) $P(g_42T\text{-}T)$ co-mixed with F_4TCNQ, (b) P3HT sequentially doped with F_4TCNQ, (c) P3HT co-mixed with F_4TCNQ, (d) P3HT spincoated from xylene corresponding to sq and (e) P3HT spincoated from chlorobenzene corresponding to co.

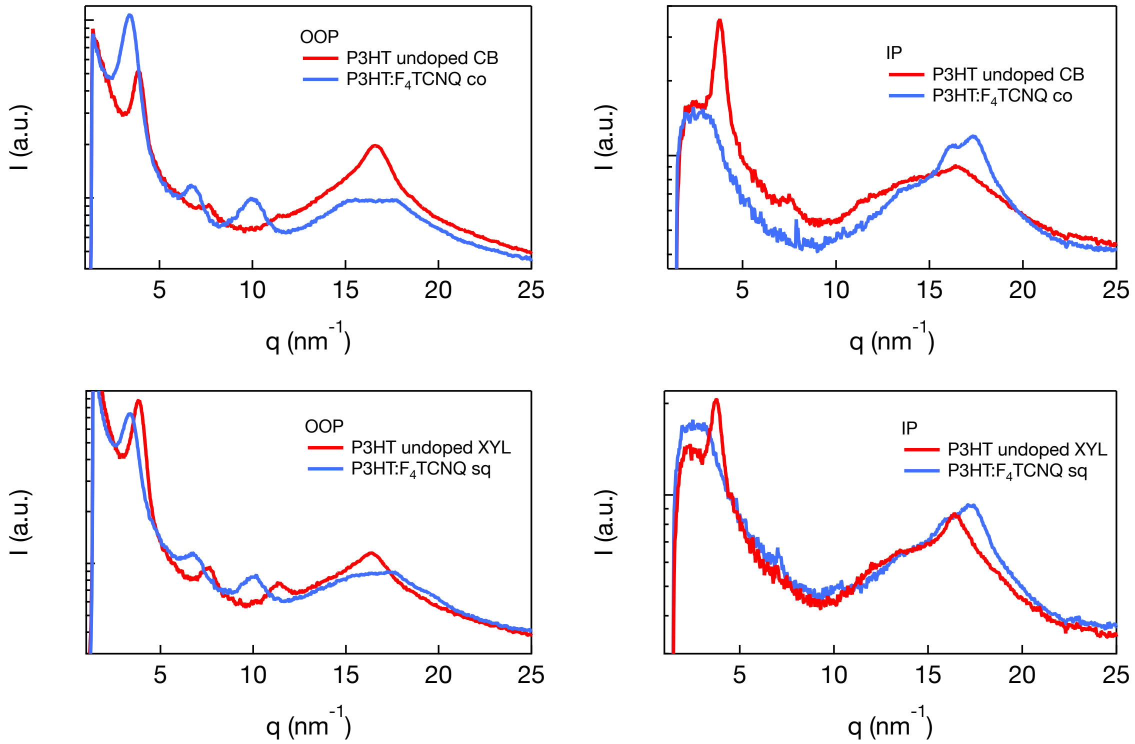

Figure S2. Linecuts from out-of-plane (OOP) and in-plane (IN) GIWAXS data of the undoped and doped P3HT films. For co-processing (and the corresponding undoped film), chlorobenzene (CB) was used as a casting solvent, while xylene (XYL) was used for sequential doping.

$P(g_42TT){:}F_4TCNQ$

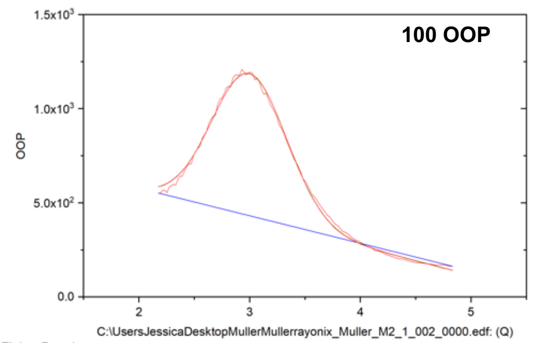

Fitting Results

Peak Index	Peak Type	Area Intg	FWHM	Max Height	Center Grvty	Area IntgP
1	Voigt	679.52976	0.81254	778.53751	2.99598	100

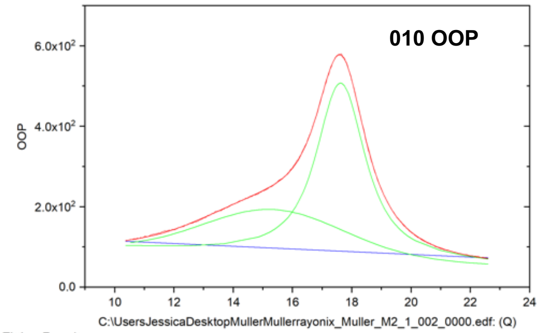

Fitting Results

Peak Index	Peak Type	Area Intg	FWHM	Max Height	Center Grvty	Area IntgP
1	Voigt	647.71054	5.53338	112.00062	15.34356	35.7678
2	Voigt	1163.16549	1.96847	433.8984	17.62174	64.2322

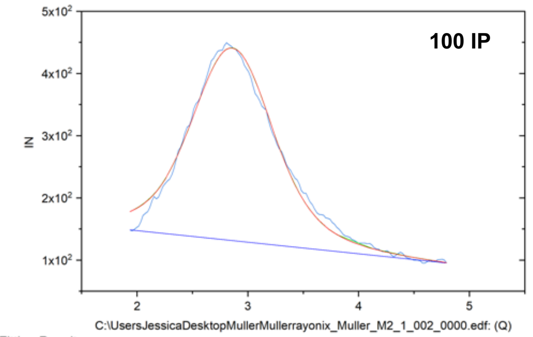

Fitting Results

Peak Index	Peak Type	Area Intg	FWHM	Max Height	Center Grvty	Area IntgP
1	Voigt	316.60418	0.87625	315.75469	2.85756	100

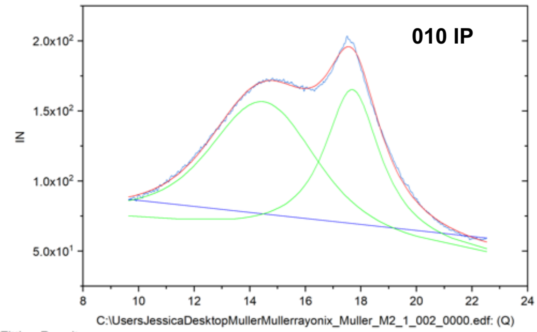

Fitting Results

Peak Index	Peak Type	Area Intg	FWHM	Max Height	Center Grvty	Area IntgP
1	Voigt	510.12443	4.65514	94.41053	14.48162	57.6595
2	Voigt	374.59381	2.49072	109.91719	17.69222	42.3404

Figure S3. Peak fits for the 100 lamellar stacking peak and the 010 π–π-stacking peak of the doped $P(g_42T\text{-}T){:}F_4TCNQ$ co film.

P3HT undoped CB

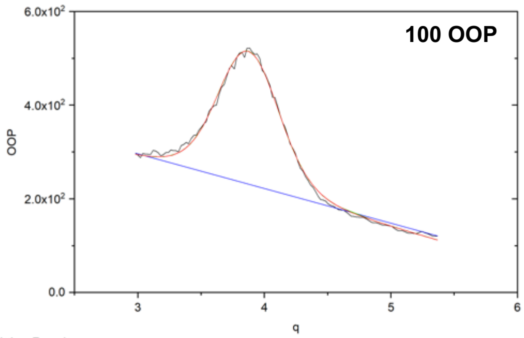

Fitting Results

Peak Index	Peak Type	Area Intg	FWHM	Max Height	Center Grvty	Area IntgP
1	Voigt	197.61441	0.58994	294.58659	3.87176	100

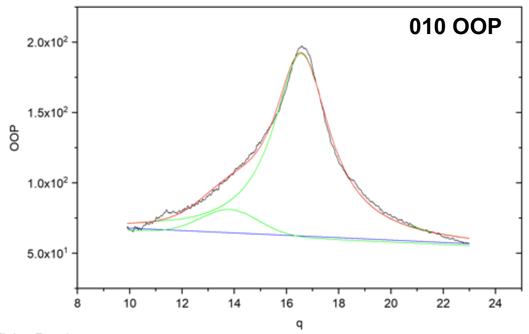

Fitting Results

Peak Index	Peak Type	Area Intg	FWHM	Max Height	Center Grvty	Area IntgP
1	Voigt	50.68276	2.64312	17.93655	13.82455	9.76493
2	Voigt	468.34574	2.604	130.84865	16.56215	90.23507

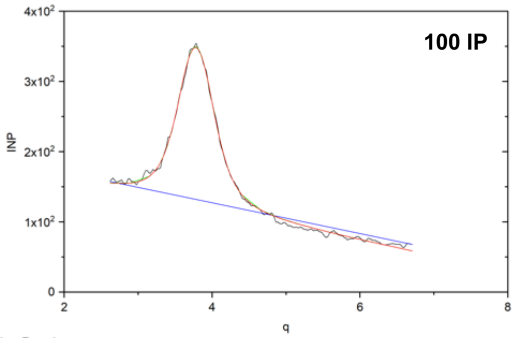

Fitting Results

Peak Index	Peak Type	Area Intg	FWHM	Max Height	Center Grvty	Area IntgP
1	Voigt	171.58079	0.5979	227.28817	3.77994	100

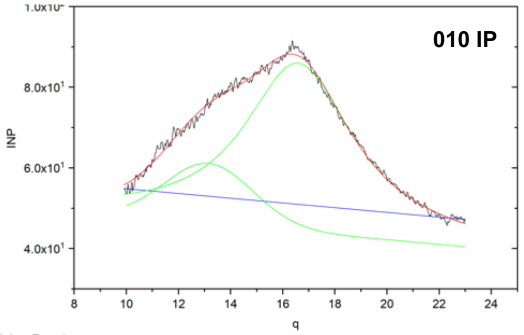

Fitting Results

Peak Index	Peak Type	Area Intg	FWHM	Max Height	Center Grvty	Area IntgP
1	Voigt	61.37439	3.98231	14.88293	13.1562	19.38551
2	Voigt	255.22492	5.10025	41.72157	16.58659	80.61449

Figure S4. Peak fits for the 100 lamellar stacking peak and the 010 π–π-stacking peak of the undoped P3HT film cast from chlorobenzene (CB).

P3HT:F_4TCNQ co CB

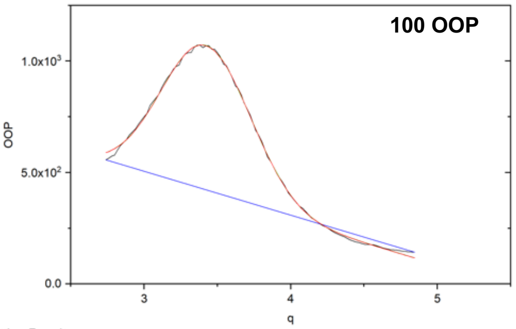

Fitting Results

Peak Index	Peak Type	Area Intg	FWHM	Max Height	Center Grvty	Area IntgP
1	Voigt	511.66462	0.7245	672.44709	3.42054	100

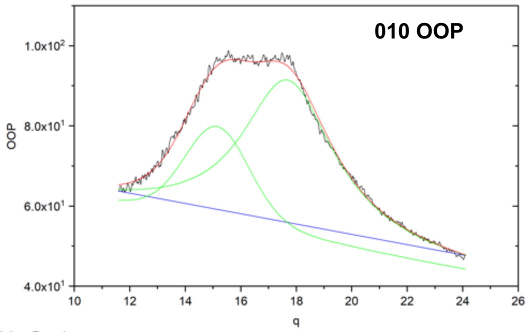

Fitting Results

Peak Index	Peak Type	Area Intg	FWHM	Max Height	Center Grvty	Area IntgP
1	Voigt	77.97266	2.85945	24.19058	15.14442	28.12436
2	Voigt	199.26973	4.06027	39.04374	17.68465	71.87564

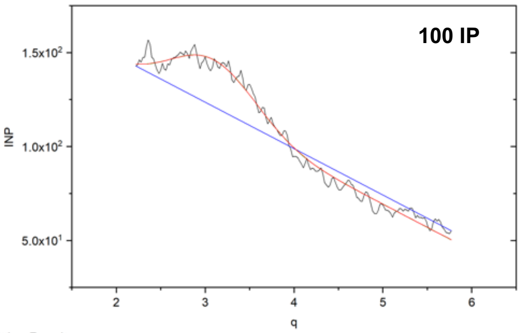

Fitting Results

Peak Index	Peak Type	Area Intg	FWHM	Max Height	Center Grvty	Area IntgP
1	Voigt	35.06579	1.14512	29.83631	3.09336	100

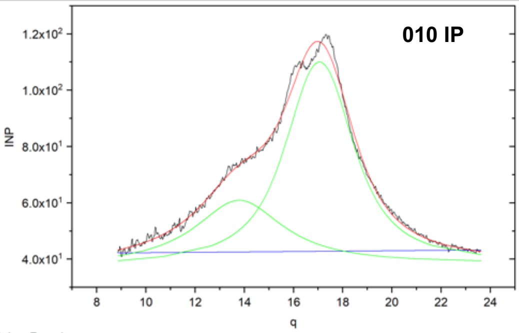

Fitting Results

Peak Index	Peak Type	Area Intg	FWHM	Max Height	Center Grvty	Area IntgP
1	Voigt	127.88593	4.3991	23.23967	13.78653	29.3802
2	Voigt	307.39335	3.29822	72.1171	17.05113	70.6198

Figure S5. Peak fits for the 100 lamellar stacking peak and the 010 π–π-stacking peak of the doped P3HT:F_4TCNQ (co) film cast from chlorobenzene (CB).

P3HT undoped XYL

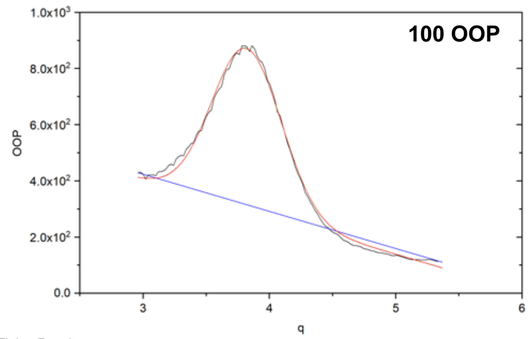

Fitting Results

Peak Index	Peak Type	Area Intg	FWHM	Max Height	Center Grvty	Area IntgP
1	Voigt	398.63326	0.65053	576.21919	3.81794	100

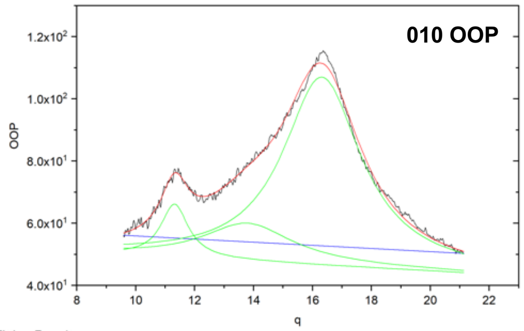

Fitting Results

Peak Index	Peak Type	Area Intg	FWHM	Max Height	Center Grvty	Area IntgP
1	Voigt	26.79122	1.13409	17.0674	11.31722	8.01356
2	Voigt	56.20884	3.71631	12.20596	13.79197	16.81272
3	Voigt	251.32331	3.21801	60.42469	16.31468	75.17372

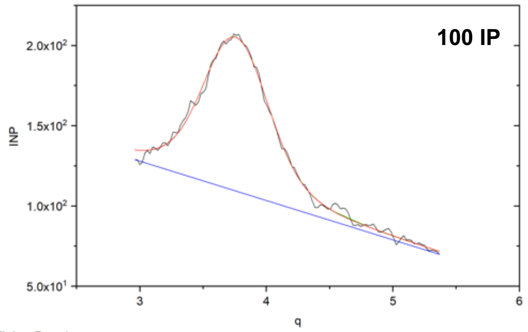

Fitting Results

Peak Index	Peak Type	Area Intg	FWHM	Max Height	Center Grvty	Area IntgP
1	Voigt	67.22811	0.62383	94.82748	3.75612	100

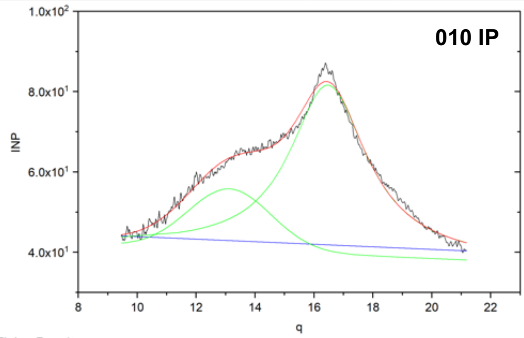

Fitting Results

Peak Index	Peak Type	Area Intg	FWHM	Max Height	Center Grvty	Area IntgP
1	Voigt	53.28543	3.31804	15.15679	13.12847	23.52215
2	Voigt	173.24753	3.17902	42.037	16.47316	76.47785

Figure S6a. Peak fits for the 100 lamellar stacking peak and the 010 π−π-stacking peak of the undoped P3HT film cast from xylene (XYL).

P3HT:F_4TCNQ sq

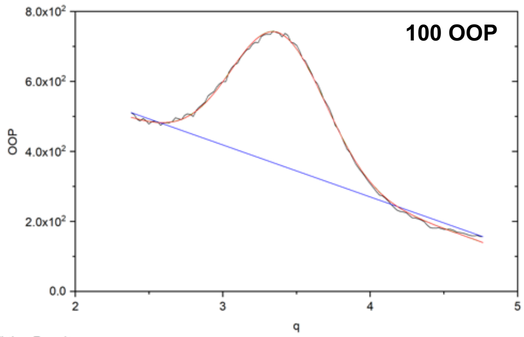

Fitting Results

Peak Index	Peak Type	Area Intg	FWHM	Max Height	Center Grvty	Area IntgP
1	Voigt	315.2281	0.75389	393.1737	3.37619	100

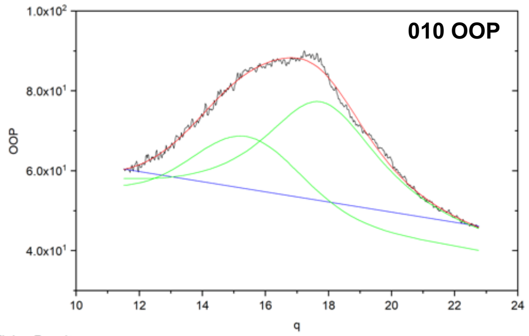

Fitting Results

Peak Index	Peak Type	Area Intg	FWHM	Max Height	Center Grvty	Area IntgP
1	Voigt	87.48001	4.17154	19.41404	15.39182	33.60009
2	Voigt	172.87648	4.77086	31.07084	17.74713	66.39991

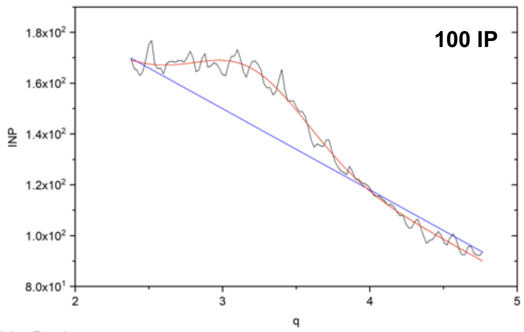

Fitting Results

Peak Index	Peak Type	Area Intg	FWHM	Max Height	Center Grvty	Area IntgP
1	Voigt	24.62571	0.91771	25.66617	3.19679	100

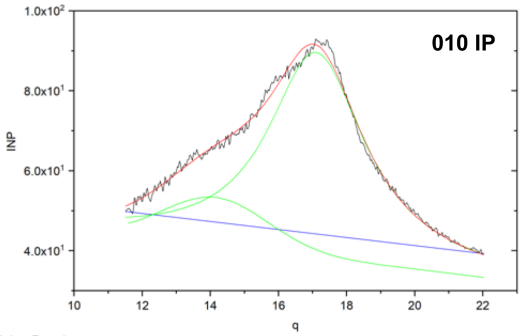

Fitting Results

Peak Index	Peak Type	Area Intg	FWHM	Max Height	Center Grvty	Area IntgP
1	Voigt	44.72535	3.6385	12.0699	14.1559	16.77657
2	Voigt	221.86874	3.45671	51.26511	17.09929	83.22343

Figure S6b. Peak fits for the 100 lamellar stacking peak and the 010 π–π-stacking peak of the doped P3HT:F_4TCNQ (sq) film cast from xylene (XYL).

Table S1: Peak position (q), peak full width at half maximum (Δq), paracrystallinity (g) and coherence length (CCL) from the GIWAXS out-of-plane (OOP) and and in-plane (IP) line cuts, from the analysis shown in Figures S3-S6. Fit errors are of the order of 10^{-2} for all parameters.

	**q (010) (nm^{-1})**	**Δq (010) (nm^{-1})**	**g (010) (%)**	**CCL (010) (nm)**	**q (100) (nm^{-1})**	**Δq (100) (nm^{-1})**	**g (100) (%)**	**CCL (100) (nm)**
**OOP**								
P3HT neat (xyl)	16.3	3.2	17.7	1.6	3.8	0.7	16.5	7.7
P3HT doped sq (xyl)	17.7	4.8	20.7	1.1	3.4	0.7	18.9	6.7
P3HT neat (cb)	16.6	2.6	15.8	1.9	3.9	0.6	15.6	8.5
P3HT doped co (cb)	17.7	4.1	19.1	1.2	3.4	0.7	18.3	7.0
**IP**								
P3HT neat (xyl)	16.5	3.2	19.4	1.3	3.8	0.6	16.3	8.1
P3HT doped sq (xyl)	17.1	3.5	17.9	1.4	3.2	0.9	21.3	5.5
P3HT neat (cb)	16.6	5.1	22.1	1.0	3.8	0.6	15.8	8.5
P3HT doped co (cb)	17.1	3.3	17.5	1.5	3.1	1.2	24.3	4.4

S3. Film thickness dependence

Table S2: Thickness and conductivities (short- and long-range) for the prepared doped polythiophene films.

	Thickness (µm)	σ_{short} (S/cm)	σ_{long} (S/cm)	Thickness (nm)	σ_{long} (S/cm)
**P(g$_4$2T-T):F$_4$TCNQ**	12	49	43	56	41
**P3HT:F$_4$TCNQ sq**	2	23	3	33	2
**P3HT:F$_4$TCNQ co**	25	4	0.2	20	0.3

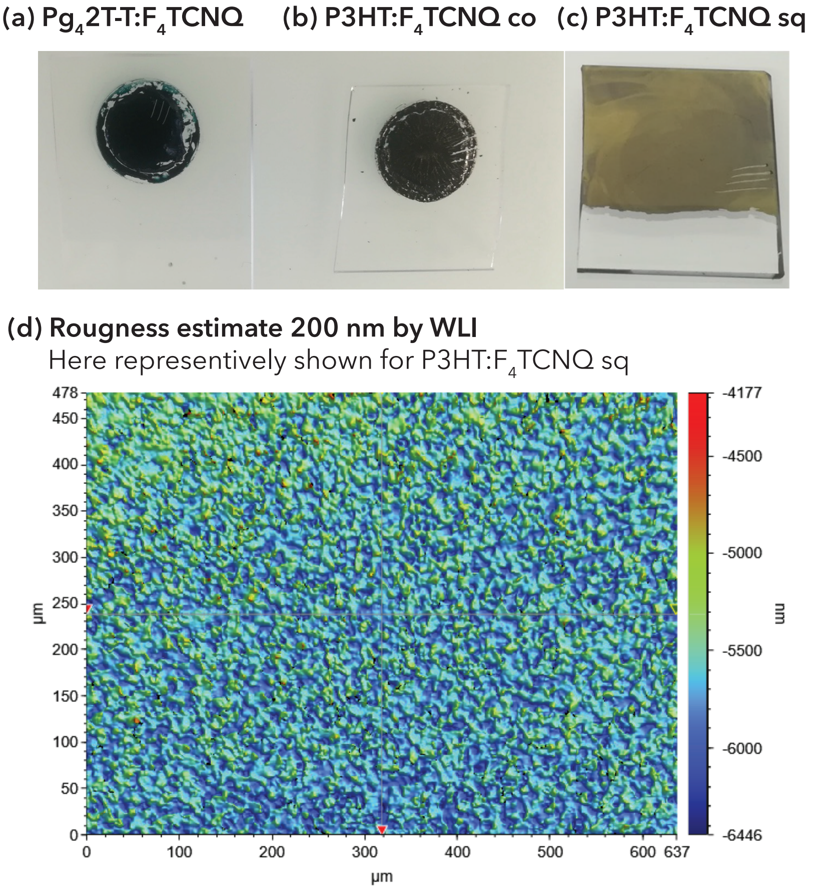

Figure S7: Pictures of the micrometer thick films (a) Pg$_4$2T-T:F$_4$TCNQ (b) P3HT:F$_4$TCNQ co and (c) P3HT:F$_4$TCNQ sq (d) White light interferometry data from a 5x magnification of film (c) representatively. The color code shows variation in thickness of 200 nm on average, which remains under 10% of the sample thickness and is a sign for high quality sample preparation.

S4. Determination of the extinction coefficients

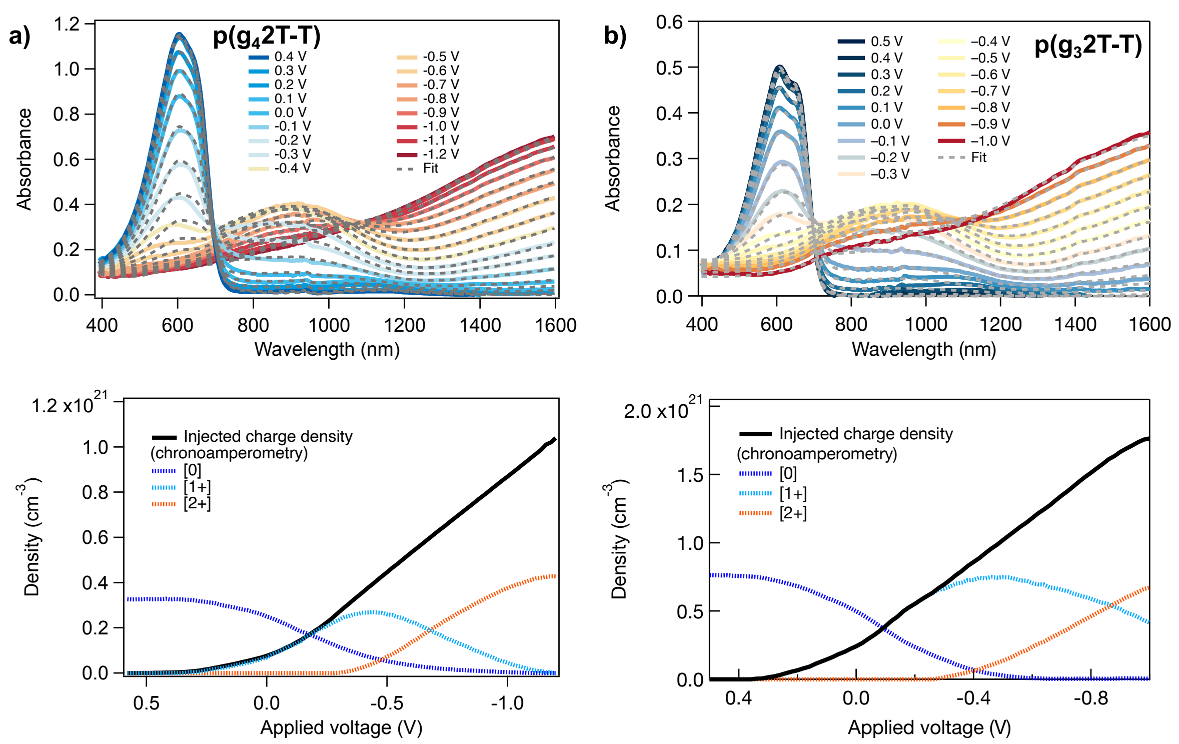

Figure S8: Spectroelectrochemical doping of (a) a P($g_4$2T-T) film and (b) a P($g_3$2T-T) film at different oxidation potentials, including the multi-variate curve resolution (MCR) fit (grey dashed curve) to the sum of spectral components (shown in Figure S9). Bottom panels: Evolution of the concentration of the species during electrochemical doping, together with the injected charge density from chronoamperometry.

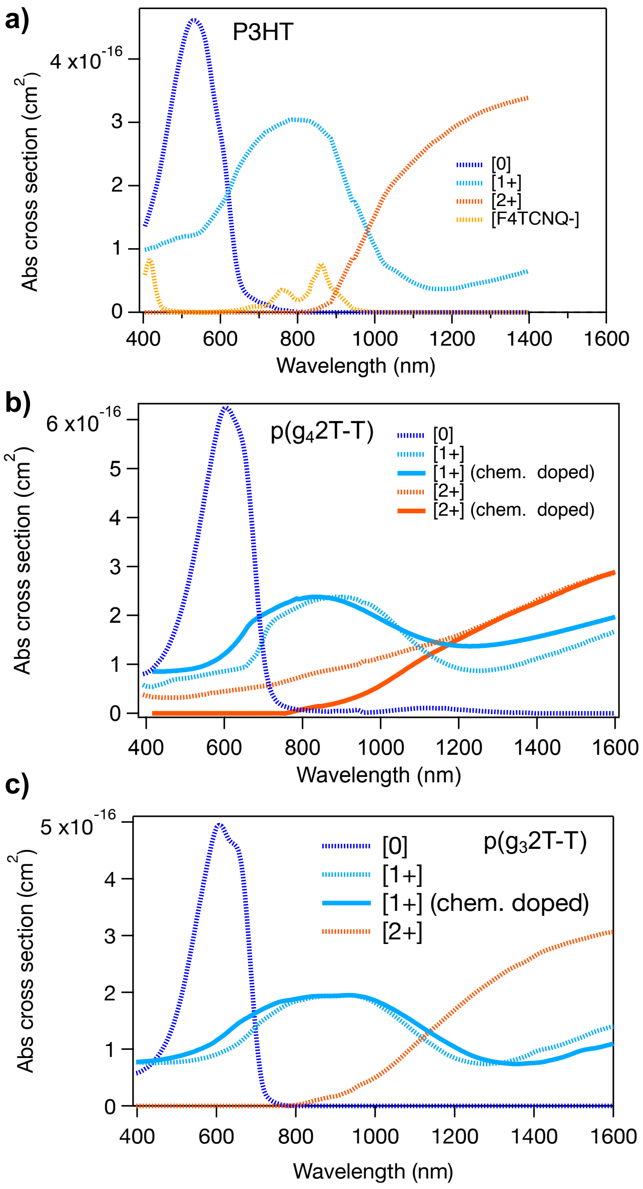

Figure S9: Absorption coefficient spectra of the neutral polymer sites [0], singly charged species [1+], doubly charged species [2+] and F_4TCNQ^- anions (from ref. 5) for (a) P3HT (from ref. 9), (b) p($g_4$2T-T) and (c) p($g_3$2T-T). The latter were obtained from the spectroelectrochemistry and chronoamperometry data shown in Figure S8 using the procedure described in ref. 9 (dotted lines). In some cases, the components needed to be slightly adapted to fit the absorbance spectra of the chemically doped samples (shown as solid lines).

S5. Species densities in the chemically doped films

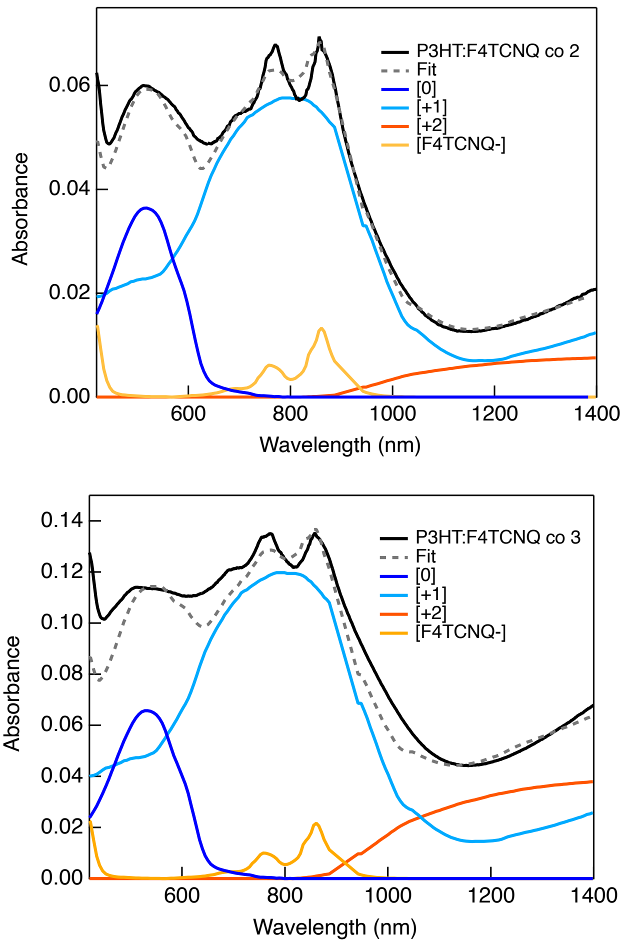

Figure S10: Steady-state absorbance spectra of additional P3HT:F_4TCNQ (co) films. The experimental spectra (black lines) were fit to the sum (dashed line) of the scaled individual spectral components (colored lines), as shown in Figure S9.

Table S3: Long-range conductivities (σ_{long}) from four-point probe measurements of several doped polythiohene films with indicated nanometer thickness, total charge density of the ionized dopant ([F_4TCNQ^-]) and of the total polymer charge density ($[Charge]_{tot}$ = [1+] + 2[2+]), absolute densities of the different oxidized species ([0], [1+] and [2+]) and ratio of the doubly to singly charged species ([2+]:[1+]).

Polymer	Dopant	Thickness (nm)	σ_{long} (*) (S cm^{-1})	Density (10^{20} cm^{-3})					[2+]:[1+]
				[F_4TCNQ^-]	$[Charge]_{tot}$	[0]	[1+]	[2+]	
P($g_4$2T-T)	F_4TCNQ co	56	41	2.4	7.3	0	4.9	1.3	0.26:1
	F_4TCNQ im (1)	543	32	0.7	3.3	0	2.3	0.5	0.22:1
	F_4TCNQ im (2)	268	23	0.7	2.9	0	2.0	0.4	0.20:1
	MB im	365	20	0	4.2	0	1.0	1.6	1.54:1
P3HT 88% RR	F_4TCNQ co (1)	20	0.2	4.0	4.8	1.2	3.6	0.6	0.16:1
	F_4TCNQ co (2)	12	0.3	3.3	4.5	1.5	3.6	0.4	0.12:1
	F_4TCNQ co (3)	30	0.4	2.2	4.7	1.1	3.0	0.9	0.28:1
	F_4TCNQ sq	33	2.0	3.6	5.9	1.3	3.9	1.0	0.25:1
	MB Im	92	60	-	7.5	0.5	3.0	2.3	0.75:1
P3HT 98% RR	F_4TCNQ co(†)	43	4	8.5	9.7	3.2	6.7	1.5	0.22:1
	F_4TCNQ sq	99	1.8	3.2	5.3	1.4	4.1	5.9	0.14:1
	F_4TCNQ im	474	5.9	2.7	2.4	1.5	2.2	0.1	0.05:1
	MB im	135	108	-	6.7	4.1	3.0	1.9	0.61:1
P($g_3$2T-T)	F_4TCNQ im	85	330	4.3	9.4	0	4.9	2.2	0.46:1
	MB im	73	140	0	13	0	3.7	4.6	1.24:1

(*) Conductivities are shown for the nanometer thick films and might slightly vary from the ones indicated in Table 2, if those were measured on micrometer thick films for comparability with the THz spectroscopy. (†)Due to film inhomogeneity, absolute values in this sample might be exaggerated, but trends and ratios are correct.

S6. Figures of merit in the complex conductivity spectra

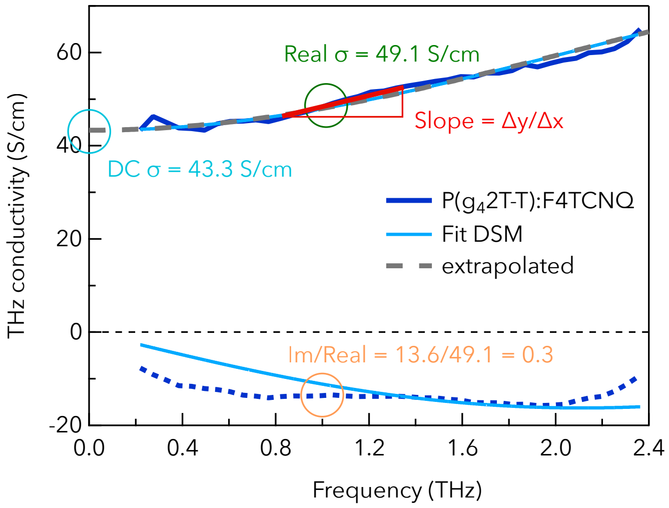

Figure S11. Determination of the observables: (i) the amplitude of the real part, (ii) the relative amplitude of the imaginary with respect to the real part, (iii) the slope of the real part and (iv) the zero-frequency (DC) conductivity from the experimental complex conductivity spectra and the extrapolation of the DSM fit.

S7. Robustness of the DSM fits

There is some flexibility in fitting the experimental data with the Drude-Smith model. For example, the scattering time (τ) and the localization parameter (c_1) have a similar effect on the shape of the curves. Also, increasing the conductive charge density (N) leads to a decreasing τ without compromising the fit. To assess the robustness of the DSM analysis and to determine the range of acceptable fit parameters, we varied N around the best fit value (Figure S2) and found the corresponding τ and c_1 values. Outside the relatively narrow depicted range, the fits rapidly deviated from the experimental curves, so we conclude that our DSM analysis is quite robust. The range of fit values is given in the main text and still shows clear trends.

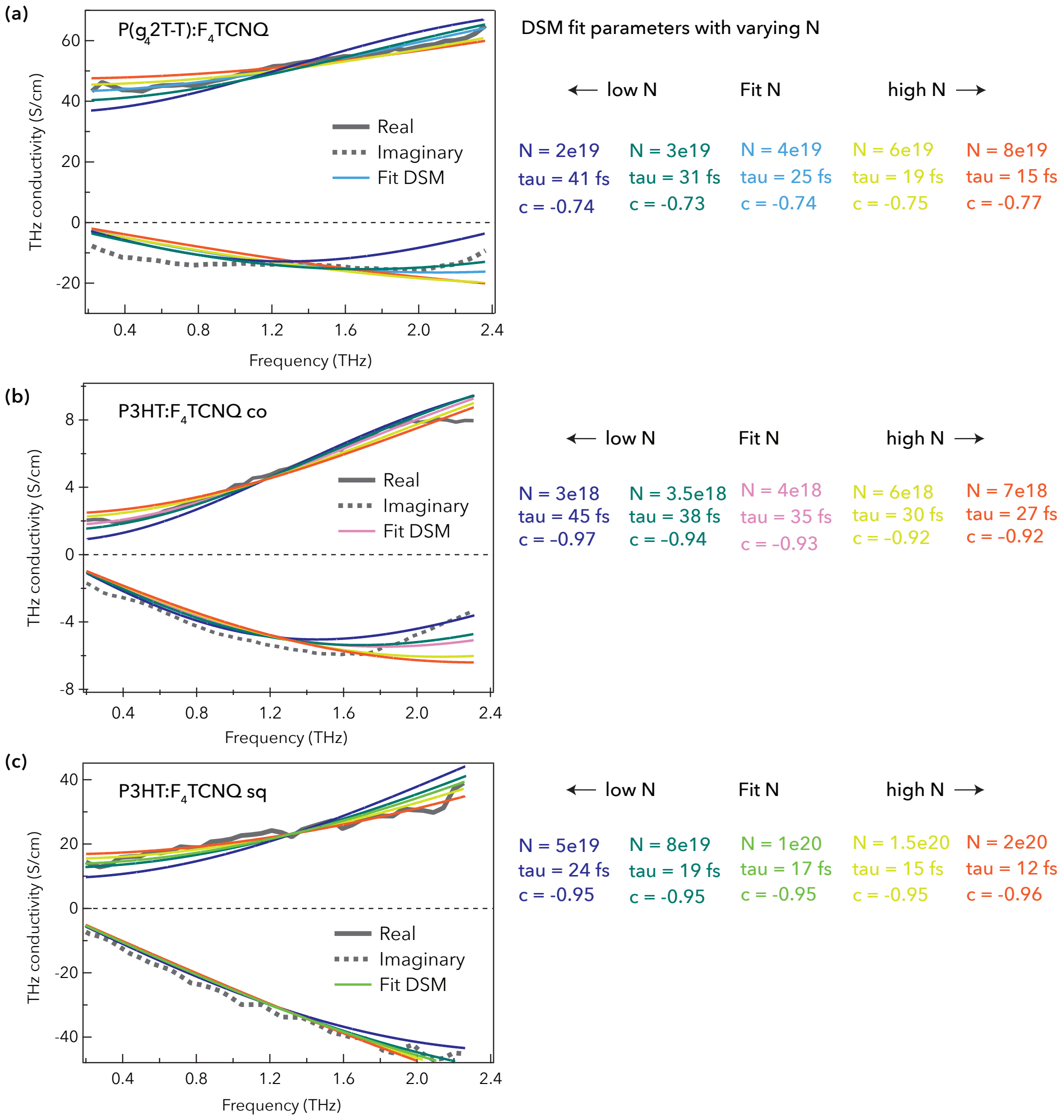

Figure S12. Drude-Smith model (DSM) fits with varying conductive charge density (N, color coded and given in cm^{-3}) and impact on the scattering time (tau) and localization parameter (c). For (a) Pg_42T-T:F_4TCNQ, (b) P3HT:F_4TCNQ co, and (c) P3HT:F_4TCNQ sq.

S8. kMC simulations

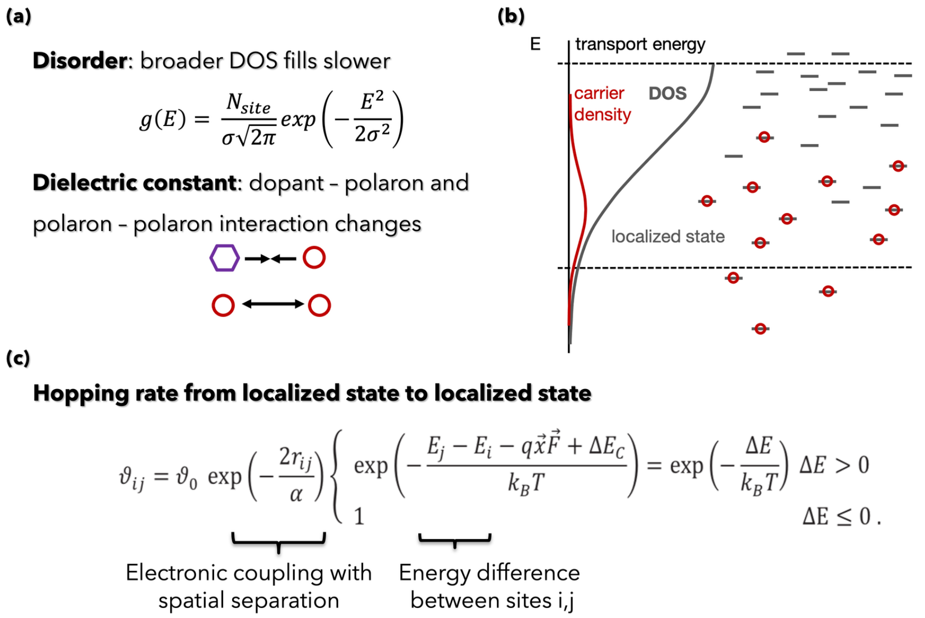

Figure S13. Schematic representation of the variable range hopping model with (a) the description of a Gaussian density of states. The density of states (DOS) at a given energy is denoted by $g(E)$, the number of sites per unit volume is denoted by N_{site} and the energetic disorder parameter or width of the DOS is denoted by σ. (b) The distribution in the DOS and (c) the effect on the hopping rate with simulated with kinetic Monte Carlo (kMC) simulations, whereby the attempt to hop frequency between sites i, j is denoted as ϑ_{ij}; the pre-exponential factor or intrinsic attempt frequency ϑ_0; the distance between sites r_{ij}; the localization length α; the energy of sites i, j is denoted as E_i and E_j respectively; the product $q\vec{x}\vec{F}$ describes the work done by the electric field $\vec{F}$ to move a polaron with elementary charge q across the distance $\vec{x}$; the Coulomb correction term ΔE_C accounts for polaron–polaron interactions; the Boltzmann constant k_B and the temperature T.

Table S4: Input parameters for the kMC simulations

Conc. of doped sites (%)	Inter-site distance (nm)	Number of neighbours to hop to	Temp. (K)	Electric field (V/m)	Attempt to hop freq. (Hz)	Energetic disorder (eV)	Dielectric constant
10	1.8	1	300	5×10^6	2×10^{13}	0.05	5
10	1.8	1	300	5×10^6	2×10^{13}	0.05	2.5
10	1.8	1	300	5×10^6	2×10^{13}	0.1	2.5

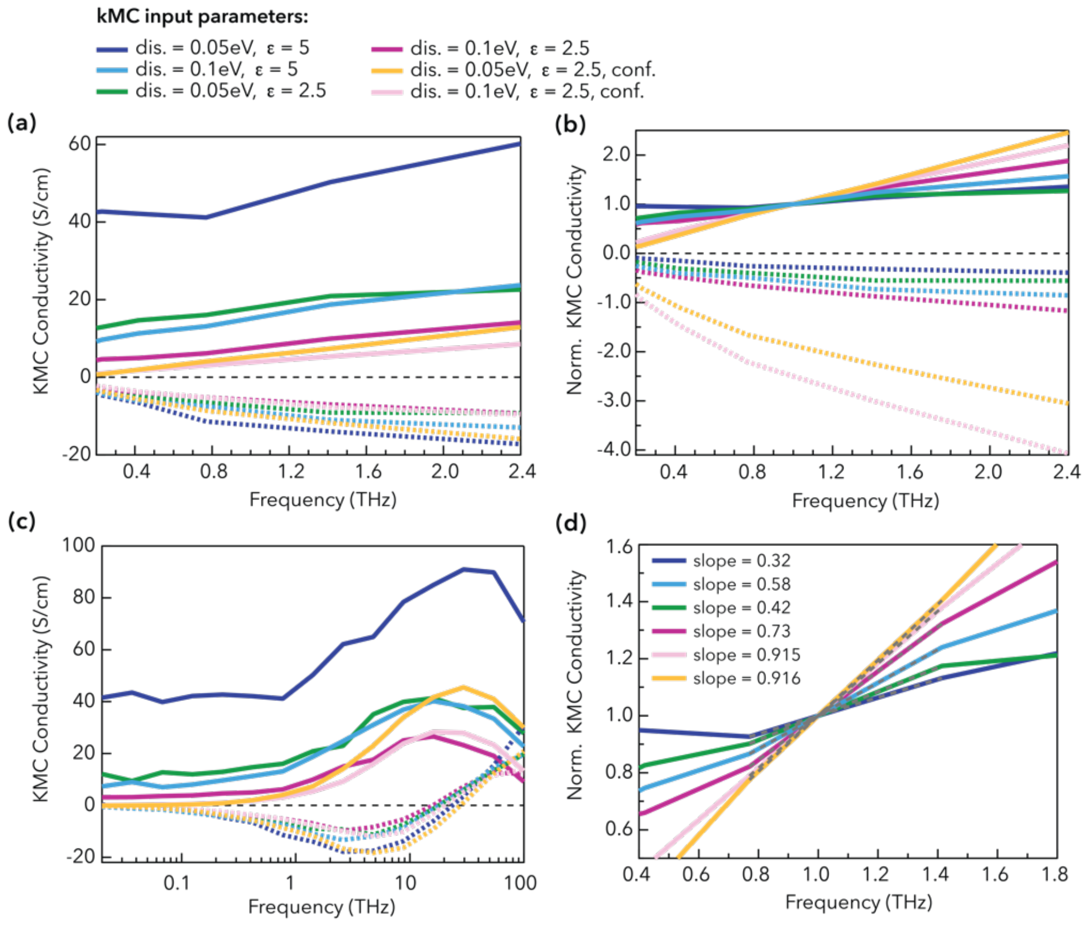

Figure S14. Complex conductivity spectra simulated by kMC for (a) a similar frequency range as for the THz experiments, (b) normalized at 1 THz, (c) the full simulated range, (d) normalized at 1 THz and zoomed-in between 0.4 and 1.8 THz to determine the slope. The kMC input parameters were the energetic disorder (dis.), the dielectric constant (ε) and the confinement to 10 sites in one direction (conf.). The conductivity (y-axis) was multiplied by a factor 20 to match the experimental data.

Table S5: Observables from the experimental and simulated complex conductivity spectra. (i) Amplitude of the conductivity at 1 THz (σ_{short}), (ii) relative amplitude of the imaginary with respect to the real part (Im/Real), (iii) slope of the real part (Slope) and (iv) extrapolated zero-frequency conductivity (σ_{DC}). The kMC simulations were run using different static energetic disorder (dis.), different dielectric constants (ε) and with or without confinement (conf.).

**Experiment**	**σ_{short} (S/cm)**	**Im/Real**	**Slope**	**σ_{DC} (S/cm)**
P($g_4$2T-T):F_4TCNQ	49	0.3	0.25	43 (DSM)
P3HT:F_4TCNQ sq	23	1.3	0.39	15 (DSM)
P3HT:F_4TCNQ co	4	1.2	0.95	2 (DSM)
**Simulation (kMC)**				
dis. = 0.05 eV, ε = 5	43	0.2	0.32	40 (kMC)
dis. = 0.05 eV, ε = 2.5	18	0.44	0.42	12 (kMC)
dis. = 0.1 eV, ε = 5	15	0.54	0.58	8 (kMC)
dis. = 0.1 eV, ε = 2.5	8	0.75	0.73	4 (kMC)
dis. = 0.05 eV, ε = 2.5, conf.	5	1.90	0.92	0 (kMC)
dis. = 0.1 eV, ε = 2.5, conf.	4	2.50	0.92	0 (kMC)

S9. Absorbance and THz data for additional materials

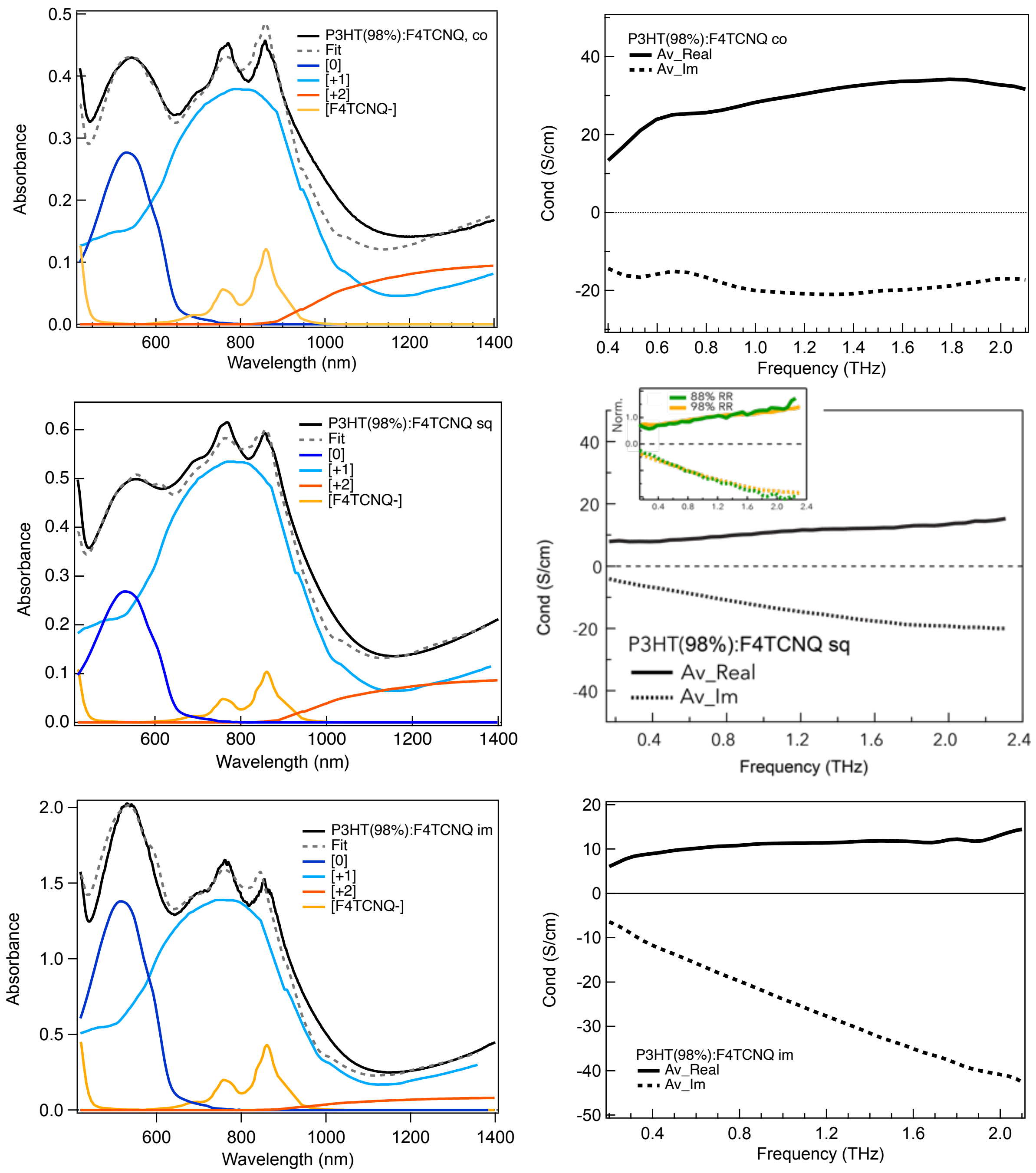

Figure S15: Left: Steady-state absorbance spectra of P3HT:F_4TCNQ films with the 98% regio-regular batch from Ossila, doped in different conditions: co-processed (co), sequential (sq) and immersed (im). The experimental spectra (black lines) were fit to the sum (dashed line) of the scaled individual spectral components (colored lines), as shown in Figure S9(a). Right: Corresponding THz complex conductivity spectra, averaged over several sample spots.

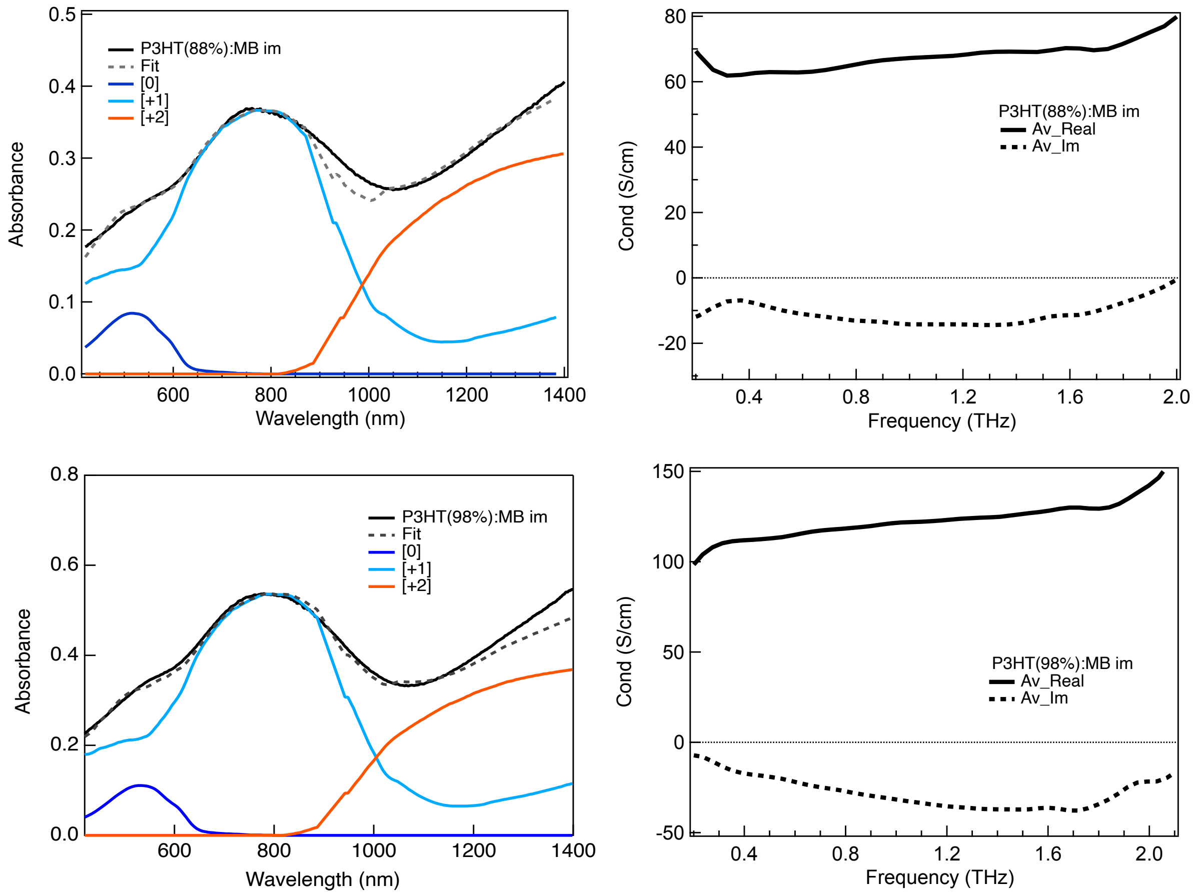

Figure S16: Left: Steady-state absorbance spectra of P3HT:MB films (immersed doping) with the 88% regio-regular batch from Solaris Chem and the 98% regio-regular batch from Ossila. The experimental spectra (black lines) were fit to the sum (dashed line) of the scaled individual spectral components (colored lines), as shown in Figure S9(a). Right: Corresponding THz complex conductivity spectra, averaged over several sample spots.

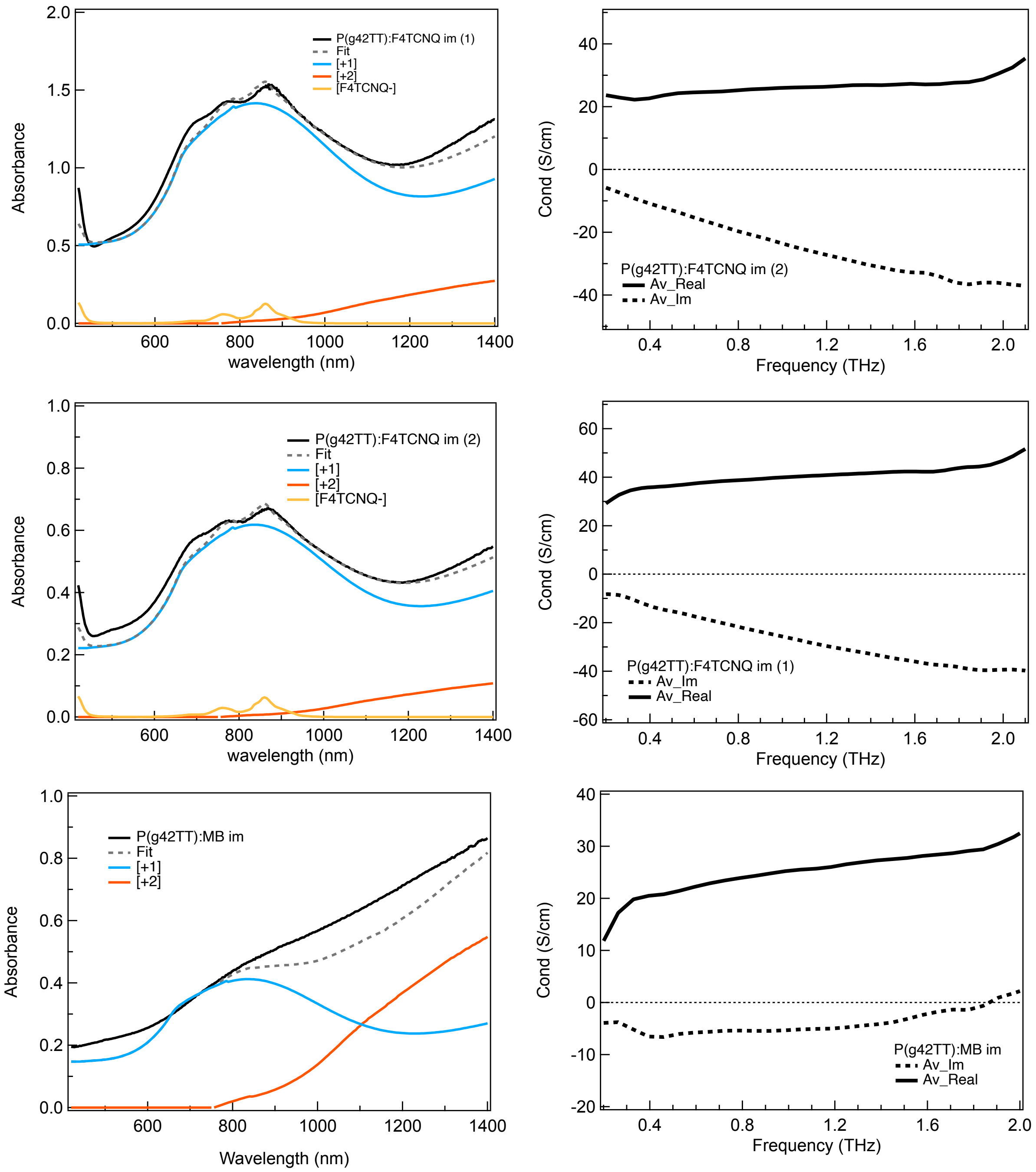

Figure S17: Left: Steady-state absorbance spectra of p(g$_4$2T-T):F4TCNQ and p(g$_4$2T-T):MB films (immersed doping). The experimental spectra (black lines) were fit to the sum (dashed line) of the scaled individual spectral components (colored lines), as shown in Figure S9(b). Right: Corresponding THz complex conductivity spectra, averaged over several sample spots.

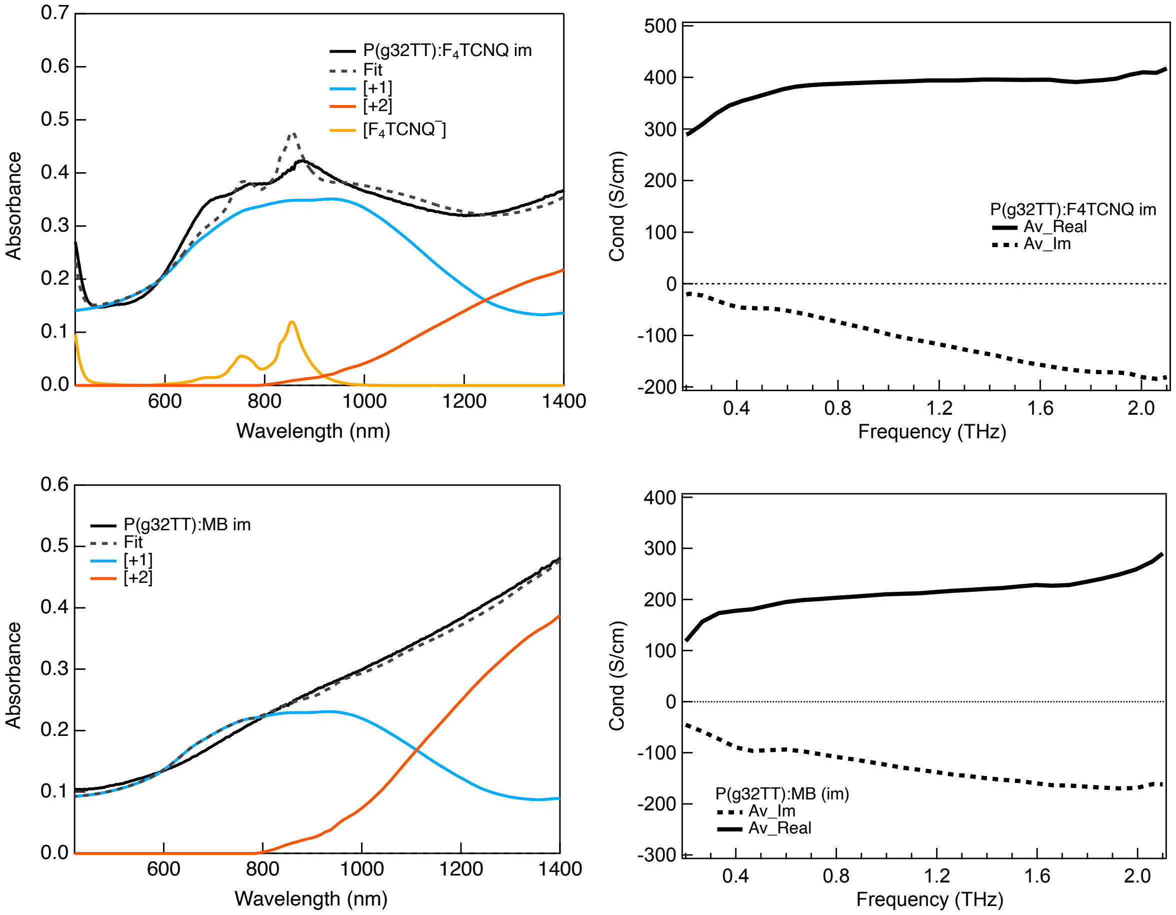

Figure S18: Left: Steady-state absorbance spectra of p(g$_3$2T-T):F$_4$TCNQ and p(g$_3$2T-T):MB films (immersed doping). The experimental spectra (black lines) were fit to the sum (dashed line) of the scaled individual spectral components (colored lines), as shown in Figure S9(c). Right: Corresponding THz complex conductivity spectra, averaged over several sample spots.

S10. SI References

1. Kroon, R.; Kiefer, D.; Stegerer, D.; Yu, L.; Sommer, M.; Muller, C. *Polar side chains enhance processability, electrical conductivity, and thermal stability of a molecularly p-doped polythiophene. Adv. Mater.* **2017,** *29*, 1700930.
2. Murrey, T. L.; Guo, K.; Mulvey, J. T.; Lee, O. A.; Cendra, C.; Bedolla-Valdez, Z. I.; Salleo, A.; Moulin, J.-F.; Hong, K.; Moulé, A. J. *Additive solution deposition of multi-layered semiconducting polymer films for design of sophisticated device architectures. J. Mater. Chem. C* **2019,** *7*, 953.
3. Zuo, G.; Abdalla, H.; Kemerink, M. *Impact of doping on the density of states and the mobility in organic semiconductors. Phys. Rev. B* **2016,** *93*, 235203.
4. Abdalla, H.; Zuo, G.; Kemerink, M. *Range and energetics of charge hopping in organic semiconductors. Phys. Rev. B* **2017,** *96*, 241202(R).
5. Yuan, D.; Plunkett, E.; Nguyen, P. H.; Rawlings, D.; Le, M. L.; Kroon, R.; Müller, C.; Segalman, R. A.; Chabinyc, M. L. *Double doping of semiconducting polymers using ion-exchange with a dianion. Adv. Funct. Mater.* **2023,** *33*, 2300934.
6. Hynynen, J.; Kiefer, D.; Yu, L.; Kroon, R.; Munir, R.; Amassian, A.; Kemerink, M.; Muller, C. *Enhanced electrical conductivity of molecularly p-doped poly(3-hexylthiophene) through understanding the correlation with solid-state order. Macromol.* **2017,** *50*, 8140.
7. Mauer, R.; Kastler, M.; Laquai, F. *The impact of polymer regioregularity on charge transport and efficiency of P3HT:PCBM photovoltaic devices. Adv. Funct. Mater.* **2010,** *20*, 2085.
8. Upreti, T.; Wang, Y.; Zhang, H.; Scheunemann, D.; Gao, F.; Kemerink, M. *Experimentally validated hopping-transport model for energetically disordered organic semiconductors. Phys. Rev. Appl.* **2019,** *12*, 064039.
9. Cavassin, P.; Holzer, I.; Tsokkou, D.; Bardagot, O.; Rehault, J.; Banerji, N. *Electrochemical doping in ordered and disordered domains of organic mixed ionic-electronic conductors. Adv. Mater.* **2023,** *35*, 2300308.
